# Harnessing computational immunology to design targeted subunit vaccines for infectious bursal disease in poultry

**DOI:** 10.3389/fbinf.2025.1562997

**Published:** 2025-04-04

**Authors:** Elijah Kolawole Oladipo, Stephen Feranmi Adeyemo, Ayomiposi Isaiah Oshoneye, Hannah Blessing Akintola, Bolatito Islam Elegbede, Tobiloba Uren Ayoomoba, Dorcas Ayomide Atilade, Omolara Omoboye Adegboye, Abuoma Elizabeth Ejikeme, Chris Olamide Balogun, Kehinde Abolade Aderibigbe, Possible Okikiola Popoola, Victoria Ajike Alabi, Boluwatife Ayobami Irewolede, Gbemi Henry Ano-Edward, Ademola Olabode Ayeleso, Helen Onyeaka

**Affiliations:** ^1^ Division of Vaccine and Pharmacotherapies Design and Development, Helix Biogen Institute, Ogbomoso, Oyo, Nigeria; ^2^ Department of Microbiology, Laboratory of Molecular Biology, Immunology and Bioinformatics, Adeleke University, Ede, Osun, Nigeria; ^3^ Department of Chemical Engineering, University of Birmingham, Birmingham, United Kingdom; ^4^ Department of Biomedical Laboratory Science, University of Ibadan, Ibadan, Oyo, Nigeria; ^5^ Department of Anatomy Pathology, Bowen University, Iwo, Osun, Nigeria; ^6^ Department of Biochemistry, Bowen University, Iwo, Osun, Nigeria; ^7^ Department of Life and Consumer Sciences, University of South Africa, Florida Park, Roodeport, South Africa

**Keywords:** infectious bursal disease virus (IBDV), immunosuppression, gastrointestinal tract, immunoinformatics, dendritic cells, immunity

## Abstract

**Introduction:**

Infectious bursal disease (IBD), caused by the infectious bursal disease Q8 virus (IBDV), is a highly contagious disease in young chickens, leading to immunosuppression with great economic importance. IBDV, a non-enveloped virus with a bipartite dsRNA genome, infects the bursa of Fabricius, causing severe gastrointestinal disease. Effective vaccines are urgently needed due to the limitations of current oral vaccines, including gastrointestinal degradation and low immunogenicity. This study designs and evaluates a multiepitope subunit vaccine using immunoinformatics.

**Methods:**

Sequences of the IBDV structural proteins VP2 and VP3 were obtained from the National Centre for Biotechnology Information) NCBI. These are structural proteins VP2 and VP3 were subjected to the Vaxijen 2.0 webserver to predict the antigenicity, ToxiPred to predict the toxicity and further analyzed to identify immunogenic epitopes of Chicken Leukocyte Antigens (CLAs) using the NetMHCpan 4.1 webserver.

**Results:**

The final vaccine construct includes 2 HTL, 21 CTL, and 7 LBL epitopes, with gallinacin-3 precursor as an adjuvant. The construct is antigenic (0.5605), non-allergenic, and non-toxic, consisting of 494 amino acids with a molecular weight of 54.88 kDa and a positive charge (pI of 9.23). It is stable, hydrophilic, and soluble. Population coverage analysis revealed a global immune coverage of 89.83%, with the highest in Europe (99.86%) and the lowest in Central America (25.01%). Molecular docking revealed strong interactions with TLR-2_1, TLR-4, and TLR-7, with TLR-7 exhibiting the highest binding affinity (−366.15 kcal/mol). Immune simulations indicated a robust immune response, with high initial IgM levels, sustained IgG, memory cell formation, and activation of T helper (Th) cells 1 and 2, Natural Killer (NK) cells, and dendritic cells, suggesting potential long-lasting immunity against IBDV.

**Discussion:**

This study presents a promising multi-epitope subunit vaccine candidate capable of effective immunization against IBDV with broad population coverage. However, further *in vivo* experimental validation is required to confirm its efficacy and safety.

## Introduction

Infectious Bursal Disease (IBD) also known as Gumboro disease, is an acute viral disease that affects young chickens (World Organisation for Animal Health, 2022) and it is caused by the infectious Bursal Disease Virus (IBDV) (Bebora et al., 2017; Mwenda et al., 2018). This has been known to cause bursal lesions, atrophy, and immunosuppression in chickens between 3 weeks and 3 months old (Mawgod et al., 2014). IBDV is a non-enveloped, icosahedral virus containing double-stranded RNA with a bisegmented genome belonging to the Avibirnavirus genus and Birnaviridae family (Jackwood et al., 2018). The bipartite dsRNA genome (segment A and segment B) of the IBDV is packaged into a single virus particle with a diameter of roughly 70 nm. There are five proteins present in IBDV, which are commonly known as the (Viral Protein 1) VP1 of 90Kd), VP2 of 40Kd, VP3 of 35Kd, VP4 of 28Kd and VP5 of 21Kd showing the molecular weights of the viral proteins (Orakpoghenor et al., 2020). Segment A is large and encodes VP2, VP3, VP4, and VP5, while segment B is smaller and encodes VP1 only, which is involved in capping and polymerase activities. The two main structural proteins of the virion are VP2 and VP3 (Sharma, 2000). The structural proteins of IBDV present various functions that apply to the life cycle and defense mechanism of the virus. These proteins are vital in the formation and replication of the virus. Structurally, VP2 is the most dominant protein of IBDV; it is a component of the capsid of the virus (Pascual et al., 2015). VP3 is another structural protein involved in IBDV life cycle as well as in the pathogenesis of the disease. It is a scaffolding protein that plays a crucial role in the viral capsid assembly and is requisite for the multiplication of the virus (Vakharia et al., 1993). It is demonstrated that VP3 interacts with the capsid protein of the virus VP2 in the construction of the virus capsid (Deng et al., 2021). This interaction is crucial in the cycling of the viral compound around the body in a manner that is likely to lead to replication.

The virus exhibits a strong tropism for lymphoid tissues, particularly targeting immature B-cells and T-cells in the bursa of Fabricius (BF) (Mwenda et al., 2018). It has also been shown to cause lymphoid depletion in BF in the free-living wild bird, which usually goes unnoticed or causes mild clinical signs only (AHA, 2009). After the entry of the virus into the host through ingestion of contaminated food or inhalation, IBDV may attach itself to proteins within the host’s cell membranes to enable viral entry into the cytoplasm of the infected cell. It infects T-lymphocytes and macrophages where the virus undergoes the first and second cycles of viremia. It infects the different lymphoid tissues and causes inflammation of the bursa of Fabricius. IBDV causes acute gastrointestinal disease and immunosuppression due to B-cell and T-cell dysfunctions. The nature and intensity of the infection depend on the status of immunity, the age of affected chickens, genetic dispositions, and the strain of the virus involved (Yip et al., 2012). There is, however, a classification of the strains mainly based on antigenicity or virulence. The first is the Classical IBDV, which are the original strains of IBDV that were first identified. They are generally less virulent and cause less severe disease compared to other strains. Another is the Variant IBDV which has emerged over time and is more virulent than classical strains. They can cause more severe diseases and have different antigenic properties, which can affect the efficacy of vaccines (Jayasundara et al., 2017). The classical subtype is additionally categorized into three pathotypes, which are mild, very virulent, and attenuated IBDV. The very virulent sub-type is highly virulent and can cause significant economic losses to the poultry industry, while the attenuated IBDV, which is a domesticated strain, does not cause obvious clinical signs and can protect against wild IBDV strains (Li et al., 2020).

There are several important advantages associated with the use of a subunit vaccine. These include safety where appropriate antigens are chosen, specificity, consistency, and storage stability for freeze-dried formulations, and the possibility of designing more antigens and formulations of vaccines to direct immune response towards certain epitopes and to define the type of immune response to be induced (Moyle, 2017). Applied to immunological knowledge, immunoinformatics is a branch that focuses on immunological computation and resources dedicated to immune function research. Medical, biological, and computing relevant knowledge and tools are used in immunoinformatics to suitably and correctly store and analyze the data related to the immune response of an organism and its roles (Oladipo E. K. et al., 2024). Immunoinformatics encompasses diverse data sources, integrating structural and functional bioinformatics tools to predict immunogenic epitopes and optimize vaccine design (Mills et al., 2015; Hegde et al., 2017). Various strategies such as using bioinformatics approach in determining predicted immunogenic epitopes on viral proteins like VP2 and VP3 of IBDV (Amit et al., 2011), studying the influence of age on immunocompetent of birds leading to increased prevalence of diseases and poor effectiveness of vaccines in aged birds (Haynes, 2020) may complement each other to enhance the understanding of the immune system of not only man but also animals and combat in contrast to some less predetermined pathogenesis.

IBDV remained a major cause of economic loss in the poultry industry due to high mortality rate and immunosuppression in chickens. This research aims to design a multiple-epitope subunit vaccine for the effective control of IBDV in chickens using bioinformatics tools. In our study, we aim to select and link viral epitopes that provide a long and strong immune response in chickens. Previous research that focused on designing a vaccine candidate suitable to combat IBDV present limitations, especially with regards to the prediction of specific Chicken Leukocyte Antigens (CLAs), resulting in the use of any Human Leukocyte Antigens (HLAs) as a proxy due to their functional and structural similarities (Gul et al., 2023). To resolve limitations in research, this study focuses on finding a solution to the economic decline in the poultry industry, which may lead to limited access to poultry-based protein. This study focuses on finding a solution to the selection of the epitopes by observing the best HLAs closer to that of Chicken Leukocyte Antigen (CLA). We also addressed the importance of computational tools in tackling the challenges surrounding vaccine design and development, which encourages a faster process, more efficient and precise results.

## Methodology

### Obtaining target protein sequences

A total of 4,509 VP2 and 43 VP3 protein sequences from Asia, Africa, Europe, North America, South America, and Australia were retrieved in FASTA format from the National Center for Biotechnology Information (NCBI) protein database (https://www.ncbi.nlm.nih.gov/) (Sayers et al., 2021). Using a threshold of 0.52 (Oladipo E. K. et al., 2024), the VaxiJen v2.0 server (https://www.ddg-pharmfac.net/vaxijen/VaxiJen/VaxiJen.html) (Doytchinova and Flower, 2007) was used to determine the antigenicity of the reference sequences.

### Prediction of cytotoxic T-cell lymphocytes (CTLs) epitopes

Cytotoxic T-Cell lymphocyte (CTL) epitopes were predicted using the NetMHCpan-4.1 NetMHCpan-4.1 server (https://services.healthtech.dtu.dk/service.php?NetMHCpan-4.1) (Reynisson et al., 2020) and the strong binding peptides were selected. The epitopes were screened based on the half-maximal inhibitory concentration threshold IC50 value. The length of the epitopes was set as 9mers. The epitopes for HLAs were selected based on the threshold of 0.6 which reveals that the binding affinity tends more toward positive than negative. However, the MixMHC2 pred server (http://ec2-18-188-210-66.us-east-2.compute.amazonaws.com:4000/#) (Racle et al., 2023) was used to select the highest quality MHC class I CLA alleles from the pool of the HLA alleles due to its accurate prediction of peptide bonds, and the ability to help in the presentation of antigen in different species. The antigenicity of the CTL epitopes was predicted using the Vaxijen 2.0 server (https://www.ddg-pharmfac.net/vaxijen/VaxiJen/VaxiJen.html) (Doytchinova and Flower, 2007) with the threshold set at 0.52. The AllerTop v2.0 (https://www.ddg-pharmfac.net/AllerTOP/) (Dimitrov et al., 2014) tool was used to assess the allergenicity of the epitopes while the toxicity of the epitopes was predicted using the ToxinPred 2 server (https://webs.iiitd.edu.in/raghava/toxinpred2/) (Sharma et al., 2022).

### Prediction of helper T-cell lymphocytes (HTLs) epitopes

To predict HTL epitopes that can bind to MHC class II alleles, VP2 and VP3 antigenic sequences were simultaneously input into the NetMHCIIPan-4.0 tool (https://services.healthtech.dtu.dk/services/NetMHCIIpan-4.0/1-Submission.php), and strong binding epitopes were selected using the threshold of 0.4 as a determinant for passing this prediction. This threshold is chosen because it is the default setting of the webserver. (Sanchez-Trincado et al., 2017). The obtained epitopes of HLA alleles were scanned via MixMHC2pred to determine the highest quality alleles closest to CLA i.e., *Gallus Gallus* alleles (http://ec2-18-188-210-66.us-east-2.compute.amazonaws.com:4000/#) (Racle et al., 2023). To enhance the analysis of epitopes, the epitopes of interest were not only analyzed for their antigenicity, allergenicity, and toxicity but also, for their ability to induce interferon-gamma (IFN-γ) cytokine using the IFN-γ epitope tool (http://crdd.osdd.net/raghava/ifnepitope/) (Dhanda et al., 2013a), Interleukin 4 (IL-4) using IL4pred (http://crdd.osdd.net/raghava/il4pred/predict.php) (Dhanda et al., 2013b) and Interleukin 10 (IL-10) using IL10 pred (http://crdd.osdd.net/raghava/il10pred/predict.php) (Nagpal et al., 2017).

### Prediction of linear B-cell epitopes

The ABCpred (http://crdd.osdd.net/raghava/abcpred/) (Malik et al., 2022) and BEPIpred (http://tools.iedb.org/bcell/) (Jespersen et al., 2017) servers were used to analyze the antigenic consensus sequences to find antigens that can stimulate the B-cell immune response, which in turn can result in the formation of antibodies. The servers use an artificial neural network to predict linear B-cell epitopes (Sanchez-Trincado et al., 2017). The threshold set for the epitopes predicted from ABCpred was 0.70, while the 0.50 threshold was set for BEPIpred server with a range of 9–16 as the length of peptides. Analysis of antigenicity, toxicity, and allergenicity were carried out to determine the efficacy of these epitopes. The prediction parameters were used to filter the final B-cell epitopes.

### Prediction of conformational B-cell epitopes

Linear and conformational epitopes are the two broad classifications of the epitopes that are fixed by the B-cells. The linear form is placed in a sequence, whereas conformational epitopes are made up of long-chain amino acids (El-Manzalawy et al., 2016). The final vaccine 3D model structure was further analyzed using the ElliPro server (http://tools.iedb.org/ellipro/) (Ponomarenko et al., 2008), to observe the solvent exposure and flexibilities, as well as the linear and structural forms of the epitopes. This server predicts the antibody epitopes based on a three-step process: First, the surface of the protein was approximated with an ellipsoid, second, every residue of the protein was assigned a protrusion index (PI) and last, neighboring residues were grouped according to the PI values. Conversely, the higher values are associated with the higher solvent accessibility of the residues in question. This method was discussed in an earlier study by Oluwagbemi et al. (2022).

### Population coverage

The use of HLA alleles that are similar to chicken alleles made it possible to proceed with the prediction of the population coverage of the vaccine constructed in this study. The epitopes binding to the MHC I and MHC II molecules were subjected to the IEDB population coverage tool (http://tools.iedb.org/population/) (Bui et al., 2006) to estimate the population size that will be immune responsive to the constructed vaccine.

### Primary vaccine construction

The primary vaccine construct was designed with modification to Oladipo E. K. et al. (2024) using immunoinformatics tools that involved identifying and selecting the best HTL, CTL, and LBL epitopes. A total of 7 LBL epitopes, 3 HTL epitopes, and 21 CTL epitopes were selected based on a multistep evaluation process, incorporating antigenicity, allergenicity, toxicity, and induction of IL4, IL10, and IFN-γ by the HTL epitopes, prioritizing those with the highest immunogenic potential and lowest adverse properties. These selected epitopes were joined together using different linkers, and a gallinacin-3 precursor was used as an adjuvant to enhance the immune response. The adjuvant, which was placed at the N-terminal, was connected to the HTL epitope using GPGPG linkers, and the same was for the HTL-to-HTL connection. KK linkers were used to connect the HTL-to-LBL and intra-LBL connections. AAY linkers were used to connect LBL-to-CTL and CTL-to-CTL.

### Antigenicity, allergenicity, and toxicity of the primary vaccine construct

Following construction, the candidate construct was subjected to antigenicity, allergenicity, and toxicity evaluation to test for the ability of the construct to elicit an immunological response. Vaxijen 2.0 (https://www.ddg-pharmfac.net/vaxijen/VaxiJen/VaxiJen.html) (Doytchinova and Flower, 2007), AllerTop (https://www.ddg-pharmfac.net/AllerTOP/) (Dimitrov et al., 2014) and ToxinPred (https://webs.iiitd.edu.in/raghava/toxinpred2/) (Sharma et al., 2022) were respectively used.

### Analysis of solubility and physicochemical properties of the primary vaccine construct

The physicochemical properties of the construct were predicted using the ExPASy ProtParam server (https://web.expasy.org/protparam/) (Gasteiger et al., 2005) which gave an exposition of the physical and chemical nature of the vaccine construct. ProteinSol server (https://protein-sol.manchester.ac.uk/) (Hebditch et al., 2017) was also employed to determine the solubility of the construct, by observing a bimodal distribution of protein solubility for *Escherichia coli* proteins in cell-free expression.

### Secondary structure prediction

The vaccine’s secondary structure, which displays the connections between the amino acids, is a crucial stage because it provides details about the relationships between the amino acids in the construct (Ma et al., 2018). SOPMA (https://prabi.ibcp.fr/htm/site/web/app.php/home) (Geourjon and Deléage, 1995), a program that uses comparable predictions from several alignments to significantly improve the secondary structure prediction of the mRNA vaccine was used.

### Three-dimensional modeling, refinement, and validation

The prediction of the tertiary structure of the vaccine construct was done with the aid of AlphaFold2 (https://github.com/sokrypton/ColabFold) (Mirdita et al., 2022) and was subjected to molecular refinement to enhance the quality of the structure. The GalaxyRefne server (https://galaxy.seoklab.org/index.html) (Heo et al., 2013) was used and the bestrefined model was selected considering the GDT-HA, RMSD, MolProbity scores, clash scores, Poor rotamers, and Rama favored scores. This tool is specifically designed to enhance the local geometry of protein structures. By performing this structural refinement, we were able to increase the confidence in the quality of the predicted model. The validation of the refined model was done using the SAVES 2.0 server (https://saves.mbi.ucla.edu/) through ERRAT (Colovos and Yeates, 1993), and PROCHECK (Laskowski et al., 2012). Continuously, QMEAN4 parameter was used to examine the model quality estimates using the SWISS-MODEL’s Quality Model Energy Analysis (QMEAN) score server (https://swissmodel.expasy.org/qmean) (Benkert et al., 2011), and ProSA-web server (https://prosa.services.came.sbg.ac.at/prosa.php) was used to analyze the stereochemical properties of the model. ProSA (Protein Structure Analysis) is a powerful tool for validation of the overall model quality which indicates whether the model has features characteristic for native structures (Wiederstein and Sippl, 2007).

### Molecular docking

The interactions between the refined model of the tertiary construct with toll-like receptors (TLRs) were studied using the HDock server (http://hdock.phys.hust.edu.cn/), which supports protein-protein docking by incorporating homology search, template-based modeling, structure prediction, macromolecular docking, and biological information incorporation for robust and fast predictions (Yan et al., 2020). Three chicken TLRs, TLR-2_1 (2 type 1) with ID: Q9DD78 (Sun et al., 2021), TLR-4 (C4PCG7) (Ruan et al., 2012), and TLR-7 (C4PCM1) (Tachibana et al., 2020) were selected and their three- dimensional structures were retrieved from the UniprotKB server (https://www.uniprot.org/) (Boutet et al., 2007).

### Molecular dynamic simulation

The docked complex of the refined tertiary construct and TLR with the highest binding affinity was subjected to molecular dynamics (MD) simulation using the WebGro protein in water simulation tool (https://simlab.uams.edu/index.php) (Abraham et al., 2015), which uses the GROMACS simulation package at default set parameters except but at 50 ns of time, to study the physical basis of the structure and function of the complex, providing time-dependent perspective on molecular interactions and changes.

### Immune simulation

**FIGURE 1:**
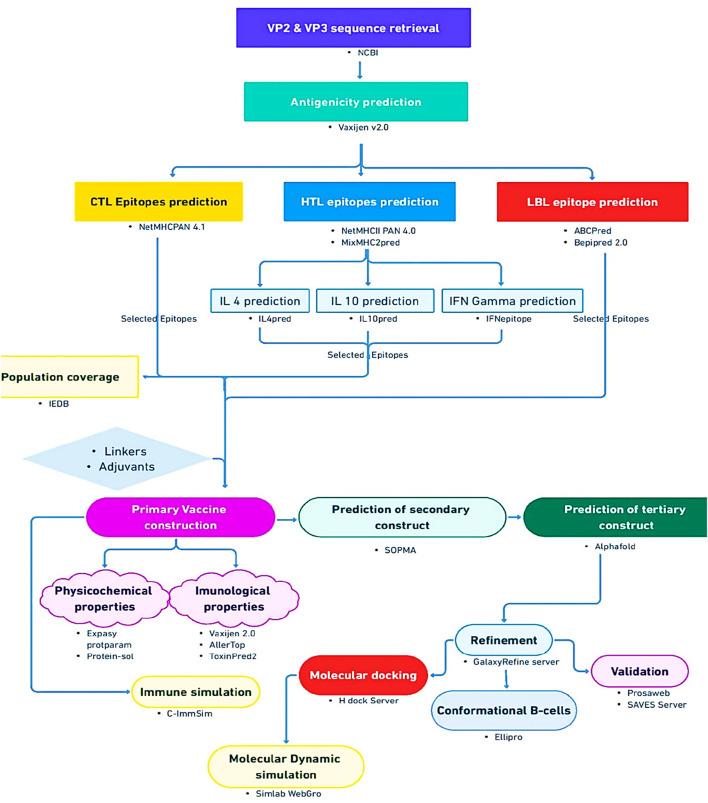
The flowchart detailing the methodology of the study.

To ascertain the interaction between the vaccine construct and the host immune system, immune simulation was carried out using default parameters of the C-ImmSim server (https://kraken.iac.rm.cnr.it/C-IMMSIM/) (Rapin et al., 2010), which uses a position-specific scoring matrix (PSSM) to assess the production of cytokines and other substances like interferon and antibodies, and in general the immune response. The general workflow of this study is shown in Figure 1.

## Results

### Obtaining target protein sequences

In developing an efficient subunit vaccine, it is paramount to find suitable antigens that induce protective immunity. Thus, out of the 4,509 VP2 and 43 VP3 protein sequences, 149 (VP2) protein sequences, as well as 15 (VP3) protein sequences from the six continents, namely, Asia, Africa, Europe, North America, South America, and Australia, were qualified based on the antigenicity scores. The data are appropriately shown in Supplementary Table S1, Supplementary Table S2, and Supplementary Table S3.

### Cytotoxic T-cell lymphocyte epitope

The 149 VP2 and 13 VP3 sequences were submitted to the NETMHC pan 4.1 server to predict CTL epitopes. However, the NETMHC pan 4.1 server is limited in the chicken alleles to be used for predicting Chicken Leukocyte antigens, therefore the 1,670 VP2 and 39 VP3 CTL epitopes of HLA alleles gotten from the server were run through MixMHC2 prediction server which helped to identify 92 VP2 CTL epitopes and 27 VP3 CTL epitopes that are of Chicken Leukocyte Antigens of alleles including Gaga-BLB1*002:01, Gaga-BLB1*012:01, Gaga-BLB2*002:01, Gaga-BLB2*012:01, and Gaga-BLB2*012:02. The selected CLA epitopes underwent antigenicity, toxicity, and allergenicity assessment, resulting in the final selection of 18 VP2 and 3 VP3 CTL epitopes as shown in Table 1.

**TABLE 1:** Final CTL epitopes based on the NETMHC pan 4.1 and MixMHC2 server.

Proteins	Epitopes	Chicken alleles	Antigenicity	Allergenicity	Toxicity
VP2	KTVWPTREY	Gaga_BLB1_002_01	Antigenic	Non -Allergen	Non-Toxic
	VTVAGVSNF	Gaga_BLB2_012_01	Antigenic	Non -Allergen	Non-Toxic
	NLMPFNIVI	Gaga_BLB2_002_01	Antigenic	Non -Allergen	Non-Toxic
	RFDPGAMNY	Gaga_BLB1_002_01	Antigenic	Non -Allergen	Non-Toxic
	DDYQFSSQY	Gaga_BLB1_012_01	Antigenic	Non -Allergen	Non-Toxic
	ITAADDYQF	Gaga_BLB2_012_01	Antigenic	Non -Allergen	Non-Toxic
	NLMPFNLVI	Gaga_BLB2_002_01	Antigenic	Non -Allergen	Non-Toxic
	VFQTSVQSL	Gaga_BLB2_002_01	Antigenic	Non -Allergen	Non-Toxic
	ITAANDYQF	Gaga_BLB2_002_01	Antigenic	Non -Allergen	Non-Toxic
	NYKFDQMLL	Gaga_BLB2_002_01	Antigenic	Non -Allergen	Non-Toxic
	YILQSNGNY	Gaga_BLB1_002_01	Antigenic	Non -Allergen	Non-Toxic
	VFKTSVESL	Gaga_BLB2_002_01	Antigenic	Non -Allergen	Non-Toxic
	VFKTNIQNL	Gaga_BLB2_002_01	Antigenic	Non -Allergen	Non-Toxic
	ILGATIYFI	Gaga_BLB1_002_01	Antigenic	Non -Allergen	Non-Toxic
	VFQTNVQNL	Gaga_BLB2_012_01	Antigenic	Non -Allergen	Non-Toxic
	SYKFDQMLL	Gaga_BLB2_002_01	Antigenic	Non -Allergen	Non-Toxic
	NLMPFNVVI	Gaga_BLB2_002_01	Antigenic	Non -Allergen	Non-Toxic
	DGNYKFDQM	Gaga_BLB1_002_01	Antigenic	Non -Allergen	Non-Toxic
VP3	KVYEINHGR	Gaga_BLB1_002_01	Antigenic	Non -Allergen	Non-Toxic
	GRGPNQEQM	Gaga_BLB1_002_01	Antigenic	Non -Allergen	Non-Toxic
	GPSPGQLKY	Gaga_BLB2_012_01	Antigenic	Non -Allergen	Non-Toxic

### Helper T-cell lymphocyte epitope

NetMHCII Pan 4.1 predicted HTL of Human leukocyte antigens (HLAs) for VP2 and VP3 epitopes, resulting in 2,447 strong bind epitopes. These epitopes of HLA alleles were run via MixMHC2pred to determine the highest quality alleles closest to CLAs i.e., *Gallus Gallus* alleles. After the subjection to the epitope to interferon, interleukin-inducing features, antigenicity, toxicity and allergenicity prediction, 7 VP2 epitopes and 1 (VP3) epitope were identified as the most promising HTL epitope candidates for the final vaccine design, and are highlighted in Table 2.

**TABLE 2:** Final HTL epitopes based on the NETMHCII pan 4.1 and MixMHC2 server.

Proteins	Epitopes	Chicken alleles	IL-4	IL-10	IFN-γ	Antigenicity	Allergenicity	Toxicity
VP2	RPRVYTITAANDYQF	Gaga_BLB1_002_01	Inducer	Inducer	Inducer	Antigen	Non-Allergen	Non-Toxic
	ANDYQFSSQYQAGGV	Gaga_BLB1_012_01	Inducer	Inducer	Inducer	Antigen	Non-Allergen	Non-Toxic
	LIVFFPGFPGSIVGA	Gaga_BLB1_002_01	Inducer	Inducer	Inducer	Antigen	Non-Allergen	Non-Toxic
	GAHYILQSNGNYKFD	Gaga_BLB1_002_01	Inducer	Inducer	Inducer	Antigen	Non-Allergen	Non-Toxic
	QMSWSARGSRALTIH	Gaga_BLB1_002_01	Inducer	Inducer	Inducer	Antigen	Non-Allergen	Non-Toxic
	GAHYTLQSNGSYKFD	Gaga_BLB1_002_01	Inducer	Inducer	Inducer	Antigen	Non-Allergen	Non-Toxic
	DNYQFSSQYQTGGVT	Gaga_BLB1_012_01	Inducer	Inducer	Inducer	Antigen	Non-Allergen	Non-Toxic
VP3	PSPGQLKYWQNTREI	Gaga_BLB1_002_01	Inducer	Inducer	Inducer	Antigen	Non-Allergen	Non-Toxic

### Linear B-cell epitope

The ABCpred server was used to generate 2,736 (VP2) and 241 (VP3) B-cell epitopes, while the BEPIpred server was used to generate 254 (VP2) and 36 (VP3) B-cell epitopes. However, the results from each tool were tested for antigenicity, toxicity, and allergenicity analysis of which 47 (VP2) and 5 (VP3) B-cell epitopes of ABCpred passed while 24 (VP2) and 1 (VP3) B-cell epitopes of BEPIpred server passed to make a total of 71 VP2 and 6 VP3 final B-cell epitopes determined to be antigenic, non-toxic, and non-allergic. These epitopes are shown in Table 3.

**TABLE 3:** Final B-cell epitopes based on the ABCpred and BEPIpred server.

Proteins	Epitopes	Antigenicity	Allergenicity	Toxicity
VP2	NDYQFSSQYQTGGVTI, TREITQPITSIKLGIE, DNYQFSSQYKTGGVTI, TITAADNYQFSSQYKA, YQFSSQYQTGGVT, TITAADDYQFSSQYQS, TSEITQPITSIKLEIV, SLSIGGELVFQTNVQD, GSVVTVAGVSNFELIP, SFSIGGELVFQTSVQS, GLTAGTDNLMPFNIVI, SARGSRALTIHAGNYP, RFDPGAMNYTKLILSE, PEDQMSWSASGSLAVT, KNLVTEYGRFDPGAMN, NDYQFSSQYQTGGVTI, VYTITAADDYQFSSQY, MTTATNKLRPFNLVIP, AGEQMSWSASGSLAVT, LTTGIDNLMPFNLVIP, VYTITAANDYQFSSQY, LGIETSKSGGQAGDQM, ANDYQFSSQYQAGGVT, DDYQFSSQYQLGGVTI, VYTITAADDYQFSSQF, AEDQMSWSASGSLAVT, KNLITEYGRFDPGAMN, YQFSSQYQTGGVT, AGDQMSWSARGSLAVT, YQFSSQYQSGGV, VGEQMSWSASGSLAVT, YQFSSQYQAGGV, TITAADNYQFSSQYKT, DNYQFSSQYQTGGVT, TITAADDYQFSSQYQP, DNYQFSSQYQAGGV, TITAADNYQFSSQYQT, DNYQFSSQYKTGGVTI, TGEQMSWSASGSLAVT, DDYQFSSQYQAGGV, SLSIGGELVFKTSIQN, YQFSSQYQTGGVTI, GLTAGTDNLMPFNLVI, DDYQFSSQYQSGGVT, TITAADNYQFSSQYQA, ADDYQFSSQYQ, GLTAGTDNLMPFNVVI, YQFSSTYQAGGV, TSQITQPITSIKLEIV, YQFSSQYKTGGVTI, TITAADDYQFSSQYQA, VAANYGLTAGTDNLMP, TITAADDYQFLSQYQP, TSKSDGQVGEQMSWSA, SLSVGGELVFQTNVQN, SSQYQAGGV, SFSIGGELVFQTSVQG, NDYQFSSQYQAGGV, SASGSLAVTIRGGNYP, DYQFSSQYQLGGV, LGATIYFIGFDGTAVI, DYQFSSQYQAGGV, DDYQFSSQYQTGGVTI, DDYQFSSQYQAGGVT, AGDQMSWSASGSLAVT, DDYQFSSQYQ, VTSKKDGQPEDQMSWS, ADNYQFSSQYKTGGVT, TSIKLEIVTSKKDGQP	Antigen	Non-Allergen	Non-Toxic
VP3	EINHGRGPNQEQMKD, GPGAFDVNTGSNWATF, TPEWVALNGHRGPSPG, YHLAMAASEFKETPEL, AMEMKHRNPRRAPPKP, YDLAMAASEFRETPEL	Antigen	Non-Allergen	Non-Toxic

### Conformational B-cells epitopes prediction

**FIGURE 2:**
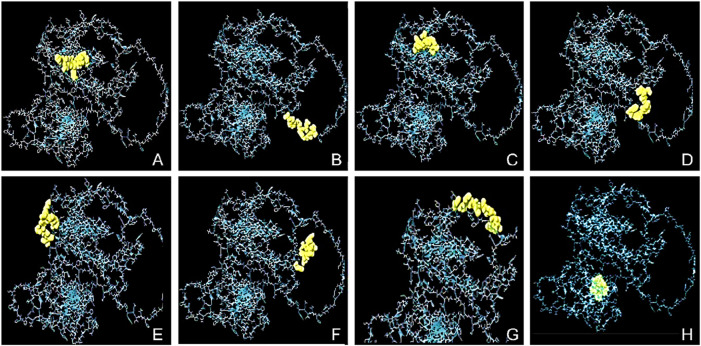
The conformational B-cell epitopes of the designed vaccine and 2D score chart. The yellow areas indicate the linear epitopes while the gray areas indicate the rest of the structure. The pI scores are as follows; **(A)** 0.891 with 8 residues, **(B)** 0.804 with 11 residues, **(C)** 0.756 with 16 residues, **(D)** 0.715 with 12 residues, **(E)** 0.683 with 8 residues, **(F)** 0.678 with 8 residues, **(G)** 0.667 with 11 residues, and **(H)** 0.562 with 8 residues.

The conformational epitopes of the B-cells were identified using the Ellipro server. Figure 2 and Table 4 summarized the positional and residue analysis of all the predicted conformational epitopes accurately.

**TABLE 4:** Predicted conformational B-cell epitopes of the final construct.

S/N	Residues	No. of residues	Score
1	A:M222, A:K223, A:D224, A:K225, A:K226, A:T227, A:P228, A:E229	8	0.891
2	A:E210, A:I211, A:N212, A:H213, A:G214, A:R215, A:G216, A:P217, A:N218, A:Q219, A:E220	11	0.804
3	A:M1, A:I3, A:V4, A:Y5, A:L6, A:L7, A:I8, A:P9, A:F10, A:F11, A:L12, A:L13, A:F14, A:L15, A:Q16, A:G17	16	0.756
4	A:A291, A:A292, A:Y293, A:I294, A:T295, A:A296, A:A297, A:D298, A:D299, A:Y300, A:Q301, A:F302	12	0.715
5	A:A328, A:Y329, A:K330, A:V331, A:Y332, A:E333, A:I334, A:N335	8	0.683
6	A:W230, A:V231, A:A232, A:L233, A:N234, A:G235, A:H236, A:R237	8	0.678
7	A:Y374, A:A375, A:A376, A:Y377, A:I378, A:T379, A:A380, A:A381, A:D382, A:D383, A:F386	11	0.667
8	A:F254, A:A255, A:A256, A:Y257, A:N258, A:L259, A:M260, A:P261	8	0.562

### Population coverage

**FIGURE 3:**
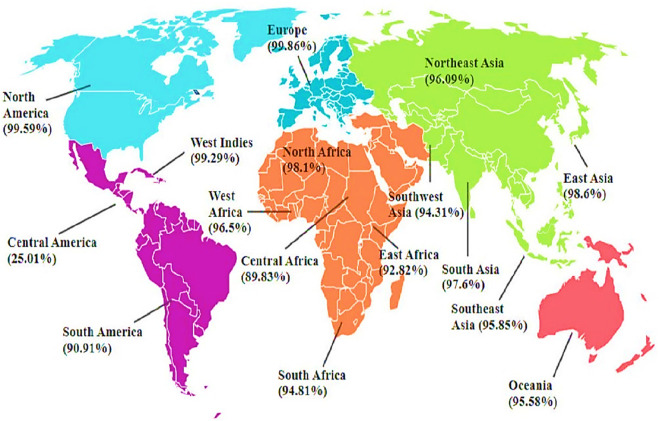
Predicted Population Coverage of Final T-cell Epitopes. The primary vaccine construct shows population coverage predictions across global regions, ranging from 99.86% in Europe (highest) to 25.01% in Central America (lowest). Other notable coverage rates include North America (99.59%), West Indies (99.29%), East Asia (98.6%), North Africa (98.1%), and South Asia (97.6%).

The results of the population coverage are shown in Figure 3. The world’s average score of the vaccine candidate is 89.83%. This demonstrates that the Infectious Bursal Disease Virus will be effectively fought off by the vaccination developed using these chosen epitopes.

### Primary vaccine construct

**FIGURE 4:**
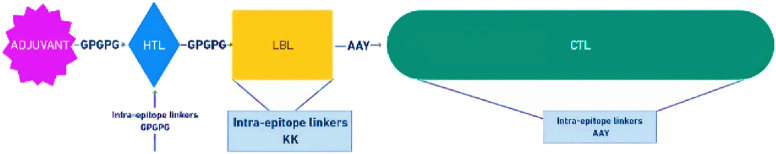
Schematic representation of the multiepitope subunit vaccine candidate, comprising of the adjuvant (purple) at the N-terminal, linked with HTL epitopes (blue) through a GPGPG linker, which also links the HTL to the LBL (yellow). The LBL epitopes is linked to the CTL epitopes (green) with the AAY linkers.

The assembled construct of the vaccine candidate contains 31 epitopes altogether (five VP2 and two VP3 LBL epitopes, two VP2 and one VP3 HTL epitopes, 18 VP2 and 3 VP3 CTL epitopes), and the gallinacin-3 precursor at the N-terminal as an adjuvant to enhance the immunogenic potential of the construct, all connected by linkers like GPGPG, KK, and AAY as shown in Figure 4.

### Antigenicity, allergenicity, and toxicity

The construct following its subjection to antigenicity, allergenicity, and toxicity evaluation shows to be antigenic with an antigenic score of 0.5605, non-allergenic, and non-toxic.

### Physicochemical properties and solubility

**FIGURE 5:**
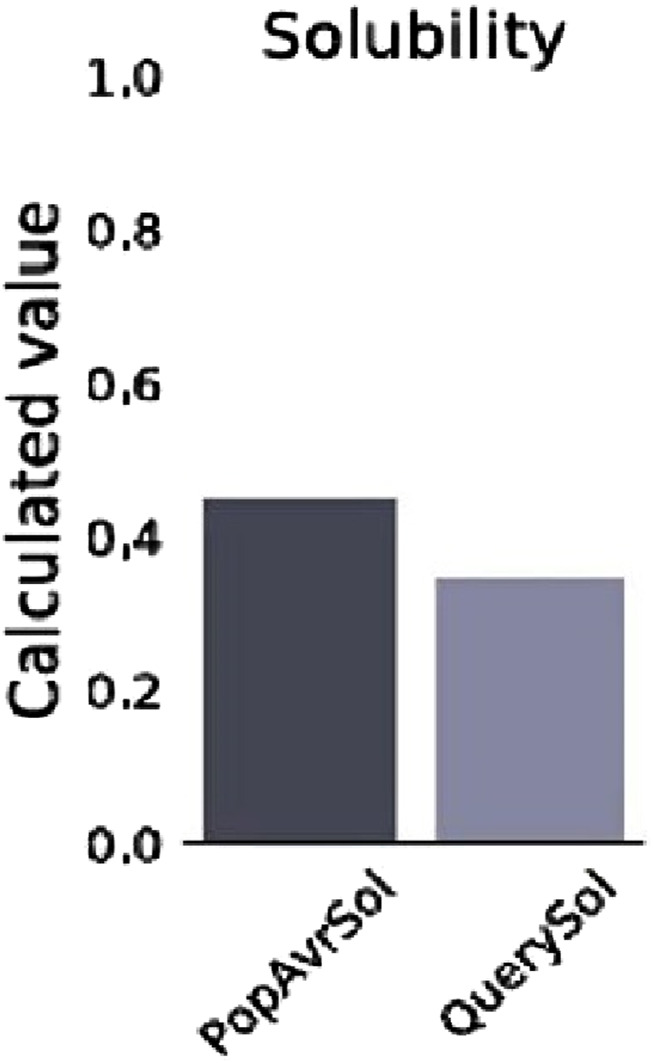
Solubility graph showing the prediction score of the vaccine construct (QuerySol) and the average soluble *Escherichia coli* protein (PopAvrSol).

The prediction of the physiochemical properties revealed that the construct has 494 amino acid residues, with a molecular weight of 54.88 kDa. The total number of negatively charged residues (Asp and Glu) is 27 and the total number of positively charged residues (Arg and Lys) is 42, this indicates that the protein is positively charged. The instability index of the vaccine construct is 27.06, thus ranking it as a stable protein as proteins with an instability index greater than 40 are regarded as non-stable. It has an aliphatic index of 73.38 indicating that the protein is stable over a wide range of temperatures and a GRAVY score of −0.187 making it hydrophilic. The solubility of the construct as shown in Figure 5 is also determined to be 0.341. The physicochemical and immunogenicity properties have been highlighted in Table 5.

**TABLE 5:** Table detailing the score for physicochemical and immunogenicity properties.

Properties	Score
Number of amino acids	494
Molecular weight	54.877kDA
Formula	C_2510_H_3761_N_645_O_714_S_15_
Total number of atoms	7645
Theoretical pI	9.23
Half-life	30 h (mammalian reticulocytes, *in vitro*) >20 h (yeast, *in vivo*) >10 h (*Escherichia coli*, *in vivo*)
Grand average of hydropathicity (GRAVY score)	−0.157
Aliphatic index	73.38
Instability index	27.06
Solubility	0.341
Antigenicity	0.5605
Allergenicity	Non-allergic
Toxicity	Non-toxic

### Secondary structure

**FIGURE 6:**
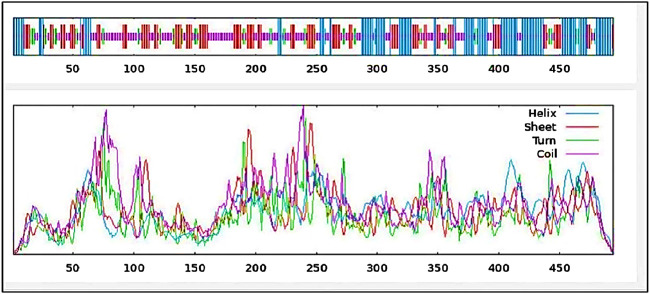
Secondary structure prediction of vaccine construct. The predicted secondary structure of the vaccine construct includes helices, sheets, turns, and coils, with their distributions visualized across the protein sequence. The top panel presents the structural arrangement, while the lower panel quantifies the frequency of each structural component.

With 29.35% alpha-helix, 25.71% extended strands, 10.53% beta-turn, and 34.41% random coils, Figure 6 and Table 6 depicts the stabilized structure of the vaccine construct and the properties of the secondary vaccine construct as a result of the analysis. This result additionally indicated that the vaccine construct possessed strong globular conformation, flexibility, and stability.

**TABLE 6:** Properties of the secondary vaccine construct.

Properties	Number of residues	Percentages
Alpha helix	145	29.35%
310	0	0.00%
Pi helix	0	0.00%
Beta bridge	0	0.00%
Extended strand	127	25.71%
Beta turn	52	10.53%
Bend region	0	0.00%
Random coil	170	34.41%
Ambiguous states	0	0.00%
Other states	0	0.00%

### Three-dimensional modeling, refinement, and validation

**FIGURE 7:**
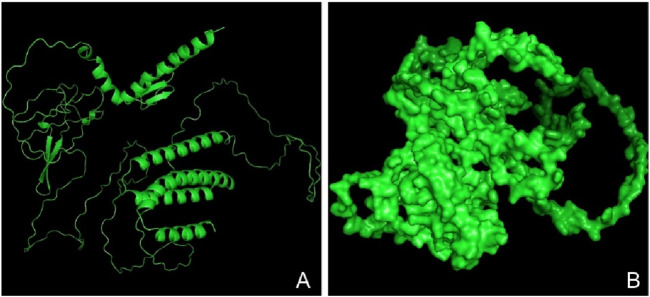
Three-dimensional structure of the vaccine construct. **(A)** A refined model of the vaccine construct (in ribbon) **(B)** Surface structure of the refined vaccine construct.

**FIGURE 8:**
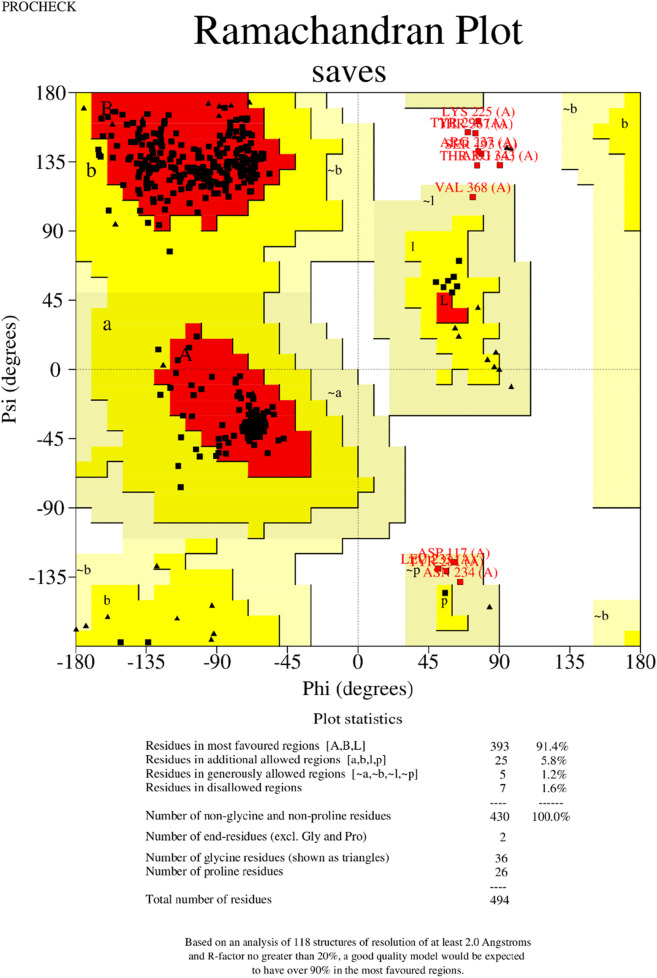
Validation of the 3−D structure. The Ramachandran plot generated by the PROCHECK shows the analysis of the residue.

**FIGURE 9:**
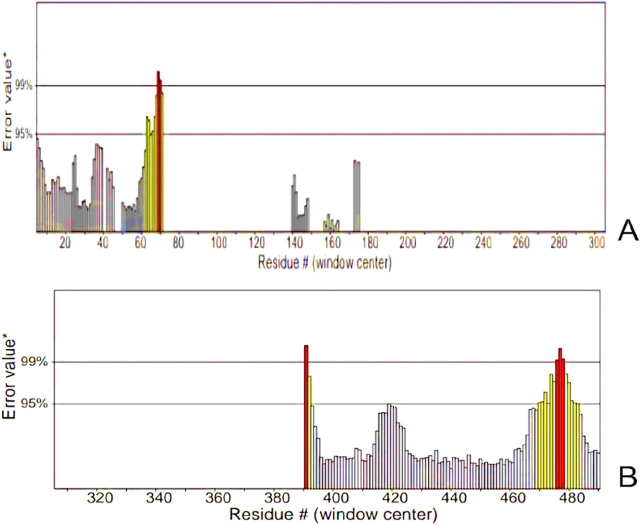
ERRAT Graphs of the Refined Model **(A)** Analysis of epitope values across the sequence revealing a pronounced peak at residue number 70, indicating a region of high antigenicity. **(B)** Residues demonstrated error values within acceptable limits. However, residues near positions 390 and 480 exhibited significantly higher error rates, surpassing the 99% error value.

**FIGURE 10:**
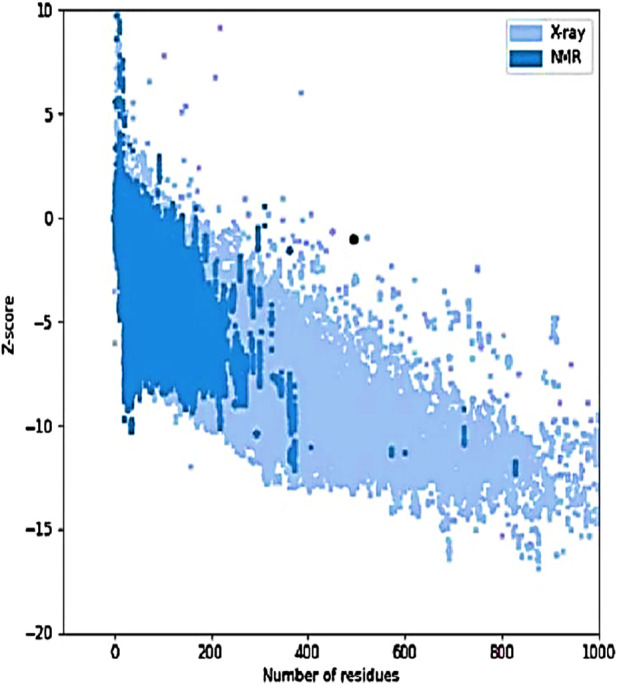
Z-score graph of the refined model. The black dot represents the IBDV vaccine model plotted against experimentally determined structures (X-ray and NMR) based on the number of residues. The model’s position within the distribution indicates acceptable overall quality and structural reliability.

**FIGURE 11:**
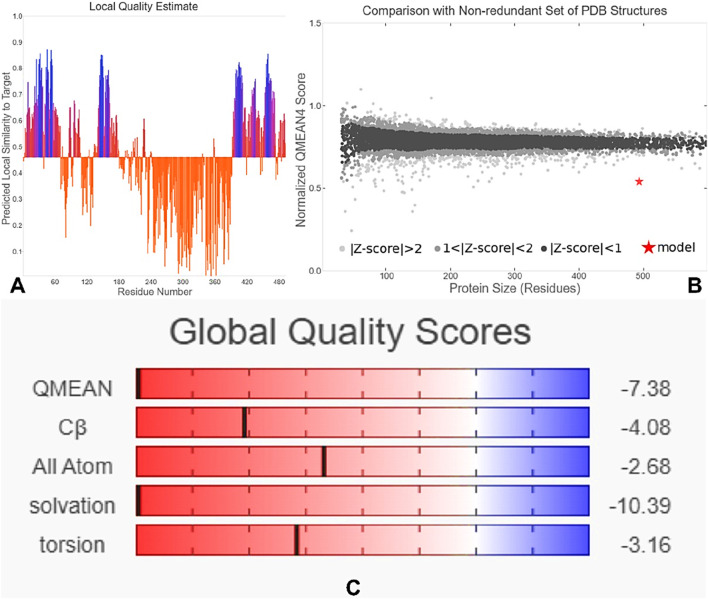
QMEAN4-based quality assessment of the 3D structure. **(A)** Local quality estimate showing residue-wise predicted similarity to high-resolution structures. **(B)** Normalized QMEAN Z-score comparing the model (red star) with PDB structures of similar length. **(C)** Global quality scores for QMEAN, Cβ, all-atom contacts, solvation, and torsion energy, indicating overall model reliability.

The tertiary structure of the IBDV-designed multiepitope construct was predicted by the AlphaFold2 server using a comparative modeling approach, while the GalaxyRefine server was employed to refine the structure. Among the five refined models, model number 1 (Figure 7) with a GDT-HA score of 0.7874, RMSD score of 0.895, MolProbity score of 1.449, clash score of 3.8, and poor rotamers of 0.3 was selected as the best structure. The validation by PROCHECK produced the Ramachandran Plot (Figure 8) showing that the most favored region of the residue is 91.4%. ERRAT shows a quality score of 86.667 (Figures 9A, B), and the Z-score obtained by the ProSA-web shows a score of −0.96 (Figure 10) signifying the high quality of the structure. The structure was also examined to have a QMEAN4 value of −7.38 as shown in Figure 11. The local quality estimate plot shows residue-wise predicted local similarity to target structures from high-resolution experimental data. Higher values (closer to 1.0) indicate better local reliability, whereas lower values reflect potentially disordered or less reliable regions. The normalized QMEAN Z-score plot compares the overall model quality against a non-redundant set of high-resolution Protein Data Bank (PDB) structures of similar lengths. The red star denotes the predicted vaccine construct, indicating its relative positioning and overall quality.

### Molecular docking

**FIGURE 12:**
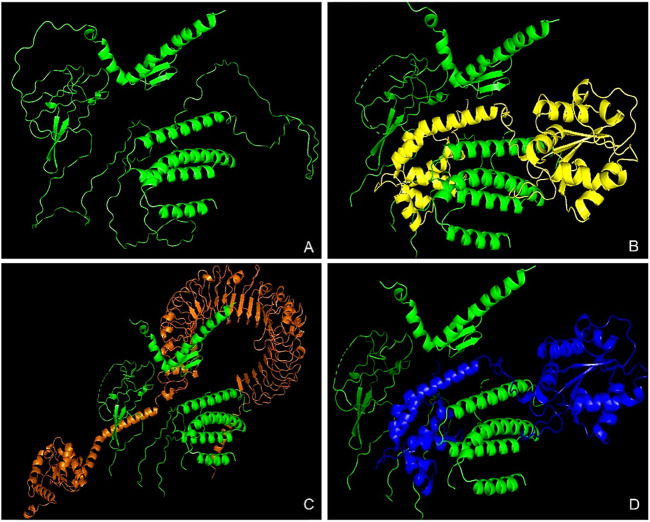
Molecular docking analysis of the vaccine construct with immune receptors. This figure presents the molecular docking results between the designed multi-epitope vaccine construct and key Toll-like receptors (TLRs). **(A)** The tertiary structure of the vaccine construct, predicted using AlphaFold, showcasing its overall 3D conformation. **(B)** Docked complex of the vaccine construct with TLR-2_1, highlighting the interaction between the vaccine and the receptor responsible for IBDV recognition. **(C)** Docked complex with TLR-4, a crucial receptor involved in innate immune activation against the viral components. **(D)** Docked complex with TLR-7, which plays a key role in viral RNA recognition and immune signaling. The colored structures represent the interacting molecules, where the vaccine construct is consistently shown in green, and receptors are displayed in distinct colors (yellow, orange, and blue) for differentiation. These docking results provide insights into the vaccine’s potential to elicit an immune response through TLR activation.

The HDock server predicted ten models of each of the docking complexes, and they were ranked based on their docking scores. The models with the lowest docking scores (model 1) of each of the docked complexes were chosen as the best models because it indicated the highest binding affinity. TLR-2_1 and the vaccine construct complex had a docking score of −366.15, TLR-4 and the vaccine construct complex had a docking score of −349.33, while TLR-7 and the vaccine construct complex had a docking score of −369.46, as shown in Table 7 and Figure 12 below.

**TABLE 7:** Summary of the docking score, confidence score, and ligand RMSD for model 1 of each docked complex.

Docked complexes	Docking score	Confidence score	Ligand RMSD (Å)
Construct and TLR-2_1	−366.15	0.9869	51.87
Construct and TLR-4	−349.33	0.9818	69.31
Construct and TLR-7	−369.46	0.9877	77.28

### Molecular dynamics simulation

**FIGURE 13:**
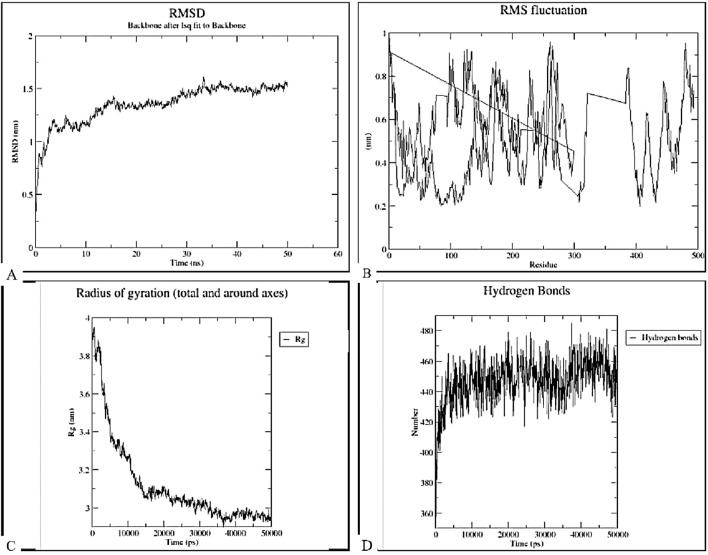
Molecular dynamics simulation analysis of the docked complex between TLR-7 and the designed vaccine construct over a 50 ns simulation period. **(A)** Root-Mean-Square Deviation (RMSD). The RMSD plot represents the structural stability of the complex over time, showing initial fluctuations followed by stabilization, indicating system equilibration. **(B)** Root-Mean-Square Fluctuation (RMSF). The RMSF plot highlights the flexibility of individual residues, identifying regions with higher fluctuations that may correspond to loop regions or binding site flexibility. **(C)** Radius of Gyration (Rg). The Rg plot demonstrates the compactness of the complex, with a decreasing trend suggesting stabilization and proper folding over the simulation time. **(D)** Hydrogen Bond Analysis. The number of hydrogen bonds maintained throughout the simulation, indicating the strength and stability of molecular interactions between the vaccine construct and TLR-7.

The stability, binding, and dynamics of the docked complex with the highest binding affinity (complex of TLR-7 and the vaccine construct) were evaluated using MD simulations of 50 ns, focusing on key parameters such as root mean square deviation (RMSD), root mean square fluctuation (RMSF), radius of gyration (Rg), and hydrogen bonding, as illustrated in Figure 13. The RMSD analysis (Figure 13A) indicated an initial increase in RMSD values, followed by fluctuations around just below 1.5 nm throughout the 50 ns simulation period. This pattern suggests that the construct-TLR7 complex reached equilibrium after an initial equilibration phase and maintained structural integrity over time. The RMSF plot demonstrated significant fluctuations across the entire range of residues, with certain peaks indicating higher RMSF values for specific residues, suggesting that these regions possess high flexibility. The radius of gyration (Rg) values ranged from 3.4 to 3.8 nm, indicating some degree of flexibility in the structural compactness of the complex. Hydrogen bonding analysis revealed fluctuations between approximately 360–480 hydrogen bonds over the 50 ns simulation. This suggests that the construct-TLR7 complex is structurally stable while maintaining functional flexibility, essential for immune activation and receptor binding.

### Immune simulation

**FIGURE 14:**
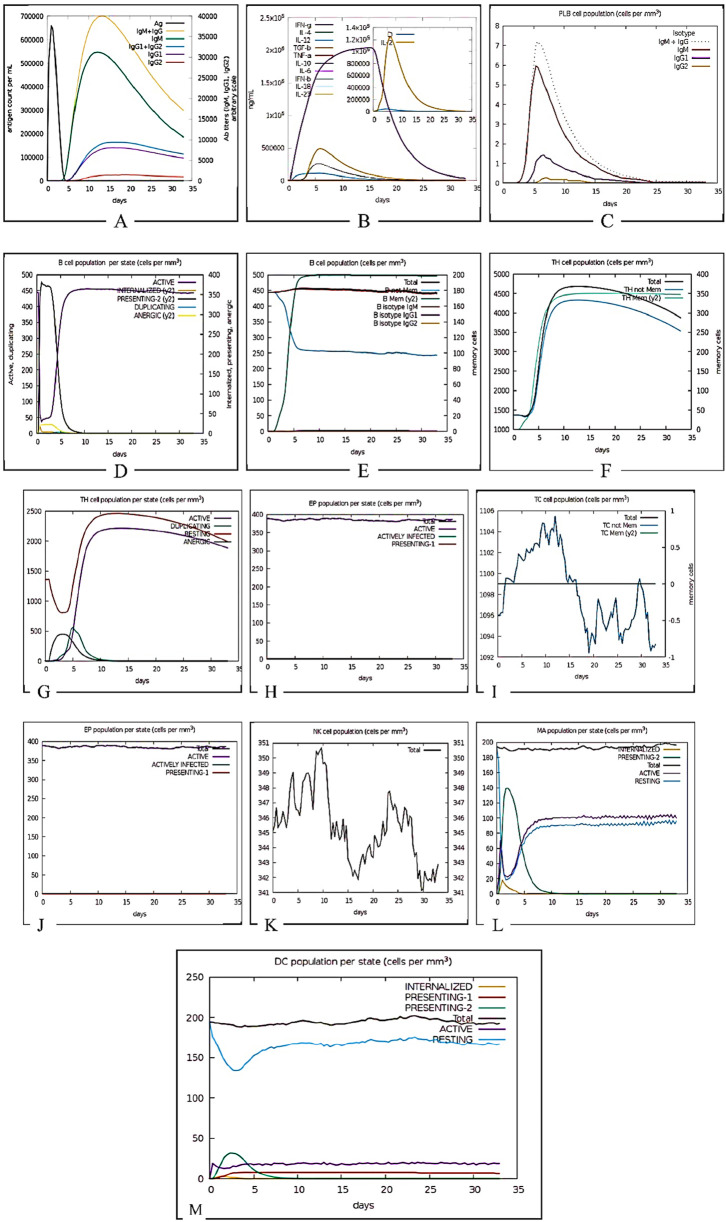
Immune simulation prediction. **(A)** Antigens, Immunoglobulins, and Immunocomplexes. This panel illustrates the dynamic interaction between antigen presence and the immune response, showing the production of different immunoglobulin (Ig) isotypes over time. The rapid increase in IgM followed by IgG subtypes indicates a typical adaptive immune response. **(B)** Concentration of Cytokines and Interleukins. This graph presents the temporal expression of key cytokines and interleukins, including IFN-γ, IL-2, IL-4, TNF-α, and TGF-β. The inset highlights a peak in IL-2 concentration, which is crucial for T-cell proliferation and differentiation. **(C)** Plasma B Lymphocyte (PLB) Population. Depicts the expansion and contraction of plasma B cells following antigen exposure. The isotype distribution of immunoglobulins produced by PLBs (IgM, IgG1, IgG2) is shown, reflecting class switching. **(D)** B Cell Population per Entity State. It tracks B cell dynamics across different functional states, including active, internalized, presenting, duplicating, and anergic states. The initial activation phase is followed by clonal expansion and antigen presentation. **(E)**Total B Lymphocyte Counts. Displays the total number of B lymphocytes, including memory B cells and isotype-specific subsets, showing the sustained presence of immune memory. **(F)**Helper T Cell Population which illustrates the expansion of total T-helper cells, including naïve and memory subsets, over the simulation period, indicating their role in orchestrating the immune response. **(G)**Helper T Cell Count per Entity State. Shows the proportion of helper T cells in different activation states, including active, duplicating, resting, and anergic cells, reflecting immune regulation. **(H)**Epithelial Cell Population per Entity State. It demonstrates epithelial cell dynamics in response to infection, with actively infected and antigen-presenting cells being highlighted. **(I)**Cytotoxic T Cell Count (Total and Memory). Tracks the expansion and differentiation of cytotoxic T cells, distinguishing between total and memory subsets. The fluctuations in memory cell numbers suggest antigen-driven responses. **(J)**Cytotoxic T Cell Count per Entity State. Shows cytotoxic T cells in active, infected, and presenting states, emphasizing their role in pathogen clearance. **(K)**Natural Killer (NK) Cell Population. Displays NK cell counts over time, with oscillations indicating periodic immune surveillance and response to infected cells. **(L)**Macrophage Populations per Entity States illustrates the distribution of macrophages in different activation states. **(M)**Dendritic Cell Population per Entity State represents dendritic cell activation states, demonstrating their crucial role in antigen presentation and initiation of adaptive immunity.

The C-ImmSim server reveals that the vaccine construct elicits a strong initial immune response evident by IgM production followed by the development of immunological memory sustaining the level of IgGs. Figure 14 shows the initial antiviral response, Th2-type response, and Th1-type response indicate some interleukin inducers which include IFN gamma, IL4 and IL12 respectively. Total B-cells and plasma B-cells initiated a strong immune response transitioning to memory cells, this indicates a typical immune reaction. Sustained levels of IgG1 and IgG2 antibodies depict successful long-term immunity. The effectiveness of the vaccine construct was also indicated by its influence on the variability in the NK cell population and activation of dendritic cells.

## Discussion

Infectious Bursal Disease (IBD) affects young chickens and has been shown to produce bursal lesions, atrophy, and immunosuppression in chickens aged three to 6 weeks (Bebora et al., 2017; Mwenda et al., 2018), posing a serious threat to the economy. As a result, there is a need to develop effective vaccinations that can aid in the fight against IBDV, particularly for the oral vaccine, which has certain issues such as gastrointestinal degradation and low immunogenicity (Lucero et al., 2021).

To address the issue, we designed a multiepitope-based subunit vaccine utilizing immunoinformatic techniques. Finding appropriate antigens that generate protective immunity is critical in the development of an effective subunit vaccine. Thus, antigenicity scores were used to qualify retrieved viral protein 2 (VP2) and viral protein 3 (VP3) sequences from six continents namely, Asia, Africa, Europe, North America, South America, and Australia.

The use of standard servers was implemented to predict the B-cell epitopes, as well as the T-cell epitopes of Human Leukocyte Antigens (HLAs). The MixMHC2pred server was employed to refine epitope selection by identifying the closest MHC class I CLA alleles from the predicted HLA alleles. The Helper T-Lymphocyte (HTL) epitopes were identified for their ability to induce key cytokines, such as IFN-γ, IL-4, and IL-10, indicating their potential for eliciting a balanced immune response.

Using standard servers (ABCpred and BEPIpred), appropriate B-cell epitopes from the targeted VP2 and VP3 proteins were found and analyzed. Using two approaches allows for cross-validation of predictions, which improves the dependability of epitope detection (Oladipo et al., 2021). The combined predictions also provide a more in-depth understanding of potential B-cell epitopes in IBDV subunit vaccine design increasing the prediction accuracy to improve the vaccination efficiency. However, seven B-cell epitopes were chosen for the final vaccine design after a satisfactory evaluation of the epitopes.

The final selection of epitopes from both VP2 and VP3 proteins for the vaccine design demonstrates a rigorous selection procedure, with the qualified epitopes having a least antigenicity score of (0.7), and a strong predicted binding to MHC class I alleles and MHC class II alleles. This ensures extensive coverage of IBDV’s antigenic landscape and viable candidates for future development (Sanchez-Trincado et al., 2017). The primary vaccine construct was manually assembled based on an immunoinformatics strategy. 7 LBL epitopes, 3 HTL epitopes, and 21 CTL epitopes were chosen to achieve high immunogenicity. These epitopes were joined using GPGPG, KK, and AAY linkers and also an adjuvant gallinacin-3 precursor was incorporated at the N-terminal to increase the immunogenicity index. Galilnacins in poultry are functional homologous of mammalian beta-defensins and are important components of the indigenous defense of a host. The expression of gallinacin in diverse tissues, such as bone marrow, bursa of Fabricius, and liver, highlights their role in bridging innate and adaptive immune responses in chickens (Hasenstein et al., 2006). Studies on the interaction between gallinacin-3 and specific Toll-like receptors (TLRs) in chickens are limited. However, there is substantial evidence suggesting that gallinacin-3 precursor (Avian β-defensin 3, AvBD3) plays a crucial role in the avian innate immune response. Zhao et al. (2001) demonstrated that gallinacin-3 expression increases significantly in the trachea following infection with *Haemophilus paragallinarum*, indicating its involvement in mucosal immunity and antimicrobial defense. A key consideration for its use as a vaccine adjuvant lies in its structural and functional similarity to human β-defensin 2 (hBD-2, DEFB4A), which is a known TLR4 and TLR2 ligand. Vora et al. (2004) showed that hBD-2 interacts with TLR4 and TLR2, triggering NF-κB activation, cytokine production, and immune cell recruitment, which are critical for enhancing both innate and adaptive immune responses. Given these parallels, gallinacin-3 may function similarly in chickens, potentially serving as a TLR4/TLR2 agonist and thereby enhancing vaccine-induced immune responses when included as an adjuvant.

A safety check was also performed to examine the construct’s antigenicity, allergenicity, and toxicity (Sharma et al., 2022). Study outcomes suggested that the construct was antigenic (score of 0.5605), non-allergenic, and non-toxic; justifying the possibility that the construct could be a good vaccine candidate. The analysed physicochemical properties of the construct revealed that it comprises 494 amino acids, has a molecular weight of 54.88 kDa, which is within the typical range for protein-based vaccines, and similar to the report of Gul et al. (2023) which has a 522 amino acid residue and a molecular weight of 55.64 kDa. The construct has an instability index of 27.06 classified as stable which demonstrates its ability to withstand harsh storage or transportation conditions, with an aliphatic index of 73.38 and a GRAVY score of −0.187 indicating its hydrophilicity, this is similar to the report of Oladipo et al. (2023) with a GRAVY of −0.261 and an aliphatic index of 75.05, demonstrating high tendency to interact with water molecule. ProteinSol analysis confirmed its solubility with a score of 0.341, supporting its practical administration. These results indicate that the vaccine construct is antigenic, stable, and immunogenic, warranting further *in vivo* validation for efficacy and safety.

The secondary structure of the vaccine construct showed 29.35% alpha-helix, 25.71% extended strands, 10.53% beta-turn, and 34.41% random coils. These depicts that the vaccine structure is stable, possess strong globular conformation and flexibility (Cheng et al., 2023). AlphaFold, a quick method for predicting protein structures was used to simulate the tertiary structure of the IBDV-multiepitope vaccine (Jumper et al., 2021; Varadi et al., 2021). The study employed the GalaxyRefine server to refine the 3-D model of the vaccine construct and SAVES server to validate the 3D model, a necessary step toward predicting a structure that is similar to the native system (Lee et al., 2019). The global distance test-high accuracy (GDT-HA) score, RMSD value, MolProbity score, clash score, rotamers score, and Rama favored score of the improved model all show good quality. The majority of the amino acid residues in the vaccine (91.4% residues) were found in the favored region, according to the improved model analyzed using Ramachandran’s plot which is similar to a study by Kumar et al. (2021). However, the vaccine’s overall quality was supported by the Z-score evaluation provided by the ProSA web service.

Following the tertiary construction of the vaccine design, the 3-D construct was docked with three toll-like receptors (TLRs). TLRs are membrane-spanning proteins that regulate cytokine production by recognizing pathogen-associated molecular patterns (Gul et al., 2023). Of the three TLRs, TLR-2_1, TLR-4, and TLR-7, a substantial interaction with a negative Gibbs-free (ΔG) value was predicted by the server. It is necessary to use the Gibbs free energy to describe the strength of an interaction that takes place in a cell under specific conditions. The more negative the Gibbs free energy value, the more energetically feasible the interaction (Yan et al., 2020). TLR-2_1 is unique to birds and is analogues to mammalian TLR-9, which recognizes unmethylated CpG DNA motifs, resulting in the activation of adaptive immune responses (Chuang et al., 2020; Keestra et al., 2010). TLR-4 though primarily known for bacterial lps induction has also been implicated in antiviral immunity in poultry by modulating inflammatory cytokine production (Keestra and Van Putten, 2008; Abdul-Careem et al., 2011). TLR-7 is crucial for detecting single-stranded RNA viruses, making it highly relevant for infectious bursal disease virus (IBDV) recognition and immune response initiation (Santana et al., 2024). The MD simulation results reveal that the construct-TLR7 complex exhibits notable stability and dynamic properties essential for its potential biological function. The RMSD analysis indicates that the complex reaches equilibrium quickly and maintains structural integrity over the 50 ns simulation period. This suggests a stable overall conformation as a low RMSD value is an indicator of a more stable protein structure (Praveen, 2024). Incorporating adjuvants and linkers can introduce additional flexibility into vaccine constructs, potentially leading to higher RMSD values. For instance, a study on a multi-epitope vaccine targeting Tropheryma whipplei incorporated an adjuvant linked to connected epitopes to boost immunogenicity and engage both innate and adaptive immunity. This design choice, while enhancing immune responses, could also contribute to increased structural flexibility and, consequently, higher RMSD values (Albekairi et al., 2022). Similarly, research on a multi-epitope vaccine against HTLV subtypes demonstrated that the incorporation of adjuvants and linkers enhanced immunogenicity but also introduced flexibility, as evidenced by molecular docking and MD simulations (Moin et al., 2023). These findings suggest that the observed RMSD values in our study may be attributed to the flexible nature of the linkers and the inclusion of adjuvants, which, while essential for eliciting robust immune responses, can lead to increased structural fluctuations.

The RMSF analysis identifies regions of high flexibility within the complex, highlighting their potential role in facilitating necessary conformational changes during interactions. This flexibility, corroborated by the Rg values (3.4–3.8 nm), suggests a balanced structural compactness that allows for functional adaptability. Hydrogen bonding analysis shows dynamic fluctuations between 360 and 480 bonds, indicating that the complex’s interactions are adaptable over time. This dynamic nature is likely important for the complex’s biological activity, particularly in immune signaling pathways where both stability and adaptability are crucial.

The immune simulation component of this research provides crucial insights into the anticipated interaction between the designed multiepitope subunit vaccine against IBDV and the host immune system, revealing the potential efficacy against IBDV. One of the unique aspects of this research was our ability to adapt the C-IMMSIM server, which is primarily designed for human immune response prediction, for our poultry vaccine construct. This was achieved by refining our pipeline during epitope mapping. Specifically, we selected epitopes for HLAs based on a threshold of 0.6, ensuring a higher binding affinity. To bridge species differences, we then utilized the MixMHC2pred server (Racle et al., 2023) to identify high-quality MHC class I and II CLA alleles respectively, which are relevant for antigen presentation in chickens. Since the initial mapping involved HLA alleles, this approach allowed us to leverage C-IMMSIM while ensuring the predicted immune response remained applicable to poultry. In the given results, the strong initial IgM response, followed by sustained IgG levels and memory cell formation, suggests that the vaccine could offer effective and long-lasting immunity (Mateus et al., 2021). The identified Th1 and Th2 responses, along with the involvement of NK cells and dendritic cells, further affirm the vaccine’s potential to invoke a multifaceted and robust immune defense. The balance of Th1 and Th2 becomes instrumental because the Th1 response characterized by IFN-γ acts in favor of cellular immunity as well as cytotoxic T-cell responses that eliminate infected cells (Hirahara and Nakayama, 2016). Th2 responses characterized by IL-4 and IL-10 act in favor of humoral immunity, stimulating B-cell maturation and antibody production. Therefore, a certain degree of Th1/Th2 balance is required to fight the IBDV since it will promote immediate clearance of the virus, which, in turn, favors some degree of immunological memory. Our simulation results demonstrate that the vaccine construct can initiate a robust and well-balanced adaptive immune response, characterized by induction of IFN-γ following Th1 stimulation and IL-4/IL-10 following Th2 stimulation (Tedla et al., 2024).

These findings prove that the vaccine construct would make an efficient vaccine candidate, unlike live attenuated vaccine which has the potential to revert to virulence and cause induction of uneven immune responses (Müller et al., 2012), our computationally designed vaccine directly incorporates highly immunogenic epitopes, which ensures targeted immune activation. The analysis found significant variation in population coverage scores between locations, indicating the heterogeneous distribution of HLA alleles and their associated immunogenic responses. Europe has the highest population coverage score of 99.86%, indicating that most chickens in the region would likely develop an effective immunological response to the vaccine according to the study of Cheng et al. (2023). This substantial coverage demonstrates the suitability of the selected epitopes for eliciting widespread immunity in European chickens. In contrast, Central America had the lowest population coverage rate, at 25.01%. This substantial gap demonstrates the difficulties of establishing universal vaccination efficacy globally. The low score indicates that the HLA alleles widespread in Central America do not match the targeted epitopes, potentially limiting the vaccine’s effectiveness in this location. East Asia (98.6%), North America (99.59%), and Oceania (95.58%) all have high population coverage scores, indicating that the vaccination would be quite effective in these locations. However, other regions’ coverage scores include Central Africa (89.83%), East Africa (92.82%), and South Africa (94.81%). With a global coverage of 89.83%, our findings align with Kardani et al. (2019), supporting the vaccine’s potential to elicit a robust immune response in diverse poultry populations.

The variation in population coverage observed from the model suggests that region specific changes to the vaccine may be needed to achieve more comprehensive and equitable immunity. The lower predicted coverage in Central America, of 25.01%, compared to Europe, of 99.86%, reveals varying regionally of Chicken Leukocyte Antigen (CLA) allele distributions that dictate epitope binding and vaccine efficacy. To address this, future vaccine should incorporate more unrestrained epitopes that bind to a wider range of MHC alleles (Schulte et al., 2023). This is a promising study, indicating that the vaccine has the potential to give large global protection against IBDV.

## Conclusion

Using immunoinformatics, a multiepitope subunit vaccine against the Infectious Bursal Disease Virus (IBDV) was designed, providing a complete and strategic strategy for combating this serious threat to poultry health. Infectious Bursal Disease (IBD) seriously impacts the poultry industry because it causes immunosuppression in chickens, increasing susceptibility to secondary illnesses and resulting in large economic losses. The immunoinformatic-driven creation of a multiepitope subunit vaccine against IBDV is a cutting-edge method that tackles the limitations of conventional vaccinations. This approach allows for a more precise selection of epitopes from chicken leukocyte antigens, increased immunogenicity, safety, and development speed. This method ensures a comprehensive and effective solution to controlling Infectious Bursal Disease, eventually protecting poultry health and increasing industry output. Future research should consider adjuvant selection beyond gallinacin-3 precursor, as adjuvants significantly influence immune response. Nonetheless, further experimental validation is necessary to confirm the vaccine’s immunogenicity, safety, and efficacy in live poultry models.

## Data Availability

The datasets presented in this study can be found in online repositories. The names of the repository/repositories and accession number(s) can be found in the article/Supplementary Material.

## Abbreviations

IBD, Infectious Bursal Disease; IBDV, Infectious Bursal Disease Virus; dsRNA, Double Stranded Deoxyribonucleic Acid; VP, Viral Proteins; BF, Bird’s bursa of Fabricius; NCBI, National Center for Biotechnology Information; CLAs, Chicken Leukocyte Antigens; HLAs, Human Leukocyte Antigens; MHC, Major Histocompatibility Complex; IL, Interleukins; ProSA, Protein Structure Analysis.
