# Supplementary material for: Harnessing computational immunology to design targeted subunit vaccines for infectious bursal disease in poultry
Source: Front Bioinform. 2025 Apr 4;5:1562997. doi: 10.3389/fbinf.2025.1562997 (PMC12006097; doi:10.3389/fbinf.2025.1562997)
Supplement: Supplementary file 1 [file Table1.docx]

**Supplementary Table 1:** Highlights of the number of retrieved VP2 protein sequences across the continents.

| **Continents** | **Number of sequences retrieved** |
| --- | --- |
| Africa | 1030 |
| Asia | 1824 |
| Europe | 603 |
| North America | 537 |
| South America | 472 |
| Australia/Oceania | 43 |
| **Total** | **4509** |

**Supplementary Table 2:** Highlights of the number of retrieved VP3 protein sequences across the continents.

| **Continents** | **Number of sequences retrieved** |
| --- | --- |
| Africa | 7 |
| Asia | 35 |
| Europe | - |
| North America | - |
| South America | 1 |
| Australia/Oceania | - |
| **Total** | **43** |

**Supplementary Table 3:** Accession list of the retrieved protein sequences

| **S/N** | **Protein** | **Accession numbers** |
| --- | --- | --- |
| 1 | VP3 | DQ912981.1, MT951626.1, MN563601.1, MN539755.1, MN539754.1, MN539753.1, MN539752.1, MN539751.1, MN539750.1, ON567385.1, JQ315182.1, JQ315181.1, JQ315180.1, JQ315179.1, JQ315178.1, JQ315177.1, JQ315176.1, JQ315175.1, JQ315174.1, JQ315173.1, JQ315172.1, JQ315171.1, JQ315170.1, JQ315169.1, JQ315168.1, JQ315167.1, JQ315166.1, JQ315165.1, JQ315164.1, JQ315163.1, JF965440.1, EF080961.1, EF080957.1, EF080955.1, EF080963.1, EF080962.1, EF080956.1, OP831905.1, OP831904.1, OP831903.1, OP831902.1, OP831901.1, OP831900.1 |
| 2 | VP3 | KC986368.1, KC986367.1, KC986366.1, KC986365.1, JX096987.1, JX096986.1, JX096982.1, JX096981.1, JX096980.1, JX096979.1, JX096978.1, JX096977.1, JX096976.1, JX096975.1, JX096974.1, JX096973.1, JX096972.1, JX096971.1, JX096970.1, JX096969.1, JX096968.1, JX096967.1, JX096966.1, JX096965.1, JX096964.1, JX096963.1, JX096962.1, JX096961.1, JX096960.1, JX096959.1, KC352669.1, KC352668.1, KC352667.1, KC352666.1, KC352665.1, KC352664.1, KC352663.1, KC352662.1, KC352661.1, KC352660.1, KC352659.1, JQ398618.1, JQ398617.1, JQ277695.1, JQ239172.1, EF544179.1, EF544178.1, EF544177.1, EF544176.1, EF544175.1, EF544174.1, EF544173.1, EF544172.1, EF544171.1, EF544170.1, AY628218.1, AY628217.1, AY628216.1, AY628215.1, AY628214.1, AB306716.1, AB306715.1, AB306714.1, AB200987.2, AB201125.1, AB201124.1, AB200988.1, AB200986.1, AB200985.1, AB200984.1, AB200983.1, AB200982.1, AB200981.1, AB200980.1, AB200979.1, AB200978.1, AB200977.1, AB200976.1, AB200975.1, LN849070.1, LN849069.1, AJ586969.1, AJ586968.1, AJ586967.1, AJ586966.1, AJ586965.1, AJ586964.1, AJ586963.1, AJ586962.1, AJ586961.1, AJ586960.1, AJ586959.1, AJ586958.1, AJ586957.1, AJ586956.1, AJ586955.1, AJ586954.1, AJ586953.1, AJ586952.1, AJ586951.1, AJ586950.1, AJ586949.1, AJ586948.1, AJ586947.1, AJ586946.1, AJ586945.1, AJ586944.1, AJ586943.1, AJ586942.1, AJ586941.1, AJ586940.1, AJ586939.1, AJ586938.1, AJ586937.1, AJ586936.1, AJ586935.1, AJ586934.1, AJ586933.1, AJ586932.1, AJ586931.1, AJ586930.1, AJ586929.1, AJ586928.1, AJ586927.1, AJ586926.1, AJ586925.1, AJ586924.1, AJ586923.1, AJ586922.1, AJ586921.1, AJ586920.1, AJ586919.1, AJ586918.1, AJ586917.1, AJ586916.1, AY819703.1, AY819702.1, AY819701.1, M97346.1 , MN478445.1, MN478444.1, MN478443.1, MN478442.1, MN478441.1, MN478440.1, MN478439.1, MN478438.1, MN478437.1, MN478436.1, MN478435.1, MN478434.1, MN478433.1, MN478432.1, MN478431.1, MN478430.1, MN478429.1, MN478428.1, MN478427.1, MN478426.1, MN478425.1, MN478424.1, MN478423.1, MN478422.1, MN478421.1, MN478420.1, MN478419.1, MN478418.1, MN478417.1, MN178132.1, OQ301987.1, OQ301986.1, OQ301985.1, OQ301984.1, OQ301983.1, OQ301982.1, OQ301981.1, OQ301980.1, MN518796.1, MN518795.1, MN518794.1, MN518793.1, MN518792.1, MN518791.1, MN518790.1, MN518789.1, MN518788.1, MN518787.1, MN518786.1, MN518785.1, MN518784.1, MN518783.1, MN518782.1, MN518781.1, MN518780.1, MN518779.1, MN518778.1, MN518777.1, MN518776.1, MN518775.1, MN518774.1, MN518773.1, MN518772.1, MN518771.1, MN518770.1, MN518769.1, MN518768.1, Z25482.1 , Z25481.1 , OR594280.1, OR594279.1, OR594278.1, OR594277.1, OR594276.1, OR594275.1, OR611974.1, OL744109.1, OL744108.1, OL744107.1, OL744106.1, OK092299.1, OK092298.1, OK092297.1, OK092296.1, OK092295.1, MT227926.1, MT138428.1, MT138427.1, MT138426.1, MT138425.1, MH676061.1, MH676060.1, MH676059.1, MK408979.1, MF988101.1, MF988100.1, MF988099.1, MF988098.1, KX078636.1, KX078635.1, KX078634.1, EU714288.1, EU714287.1, AY841902.1, AY841901.1, AY841900.1, AY512594.1, AF508177.1, AF159219.1, AF159218.1, AF159217.1, AF159216.1, AF159215.1, AF159214.1, AF159213.1, AF159212.1, AF159211.1, AF159210.1, AF159209.1, AF159208.1, AF159207.1, JN106051.1, AY321527.1, AY321524.1, AY321522.1, AY321518.1, EF529700.1, KT281984.1, FJ848772.1, DQ785174.1, DQ785173.1, DQ785172.1, DQ785171.1, Z25480.1 , OR795757.1, OR795756.1, OR795755.1, OR795754.1, OR795753.1, OR795752.1, OR795751.1, OR795750.1, OR795749.1, OR795748.1, OR795747.1, OR682619.1, OR682618.1, OR597055.1, OR597054.1, OR597053.1, OR597052.1, OR597051.1, OR597050.1, OR597049.1, OR597048.1, OR597047.1, OR597046.1, OR597045.1, OR597044.1, OR597043.1, OQ930535.1, OQ930534.1, OQ930533.1, OQ930531.1, OQ930531.1, OQ930530.1, OQ930529.1, OQ930528.1, OQ930527.1, OQ930526.1, OQ930525.1, OQ930524.1, OQ930523.1, OQ930522.1, OQ930521.1, OQ930520.1, OQ930519.1, OQ930518.1, OQ930517.1, OQ930516.1, OQ930515.1, OQ930514.1, OQ930513.1, OQ930512.1, OQ930511.1, OQ930510.1, OQ930509.1, OQ930508.1, OQ930507.1, OQ930506.1, OQ930505.1, OQ930504.1, OQ930503.1, OQ930502.1, OQ930501.1, OQ930500.1, OQ930499.1, OQ930498.1, OQ930497.1, OQ930496.1, OQ930495.1, OQ930494.1, OQ930493.1, OQ930492.1, OQ930491.1, OQ930490.1, OP727927.1, OP727926.1, OP727925.1, OP727924.1, OP727262.1, OP978089.1, OP978088.1, OP978087.1, OP978086.1, OP978085.1, OP978084.1, OP978083.1, OP978082.1, OP978081.1, OP978080.1, OP978079.1, OP978078.1, OP978077.1, OP978076.1, OP978075.1, OP978074.1, OP978073.1, OP978072.1, OP978071.1, OP978070.1, OP978069.1, OP978068.1, OP978067.1, OP978066.1, OP978065.1, OP978064.1, OP978063.1, OP978062.1, OP978061.1, OP978060.1, OP978059.1, OP978058.1, OP978057.1, OP978056.1, OP978055.1, OP978054.1, OP978053.1, OP978052.1, OP978051.1, OP978050.1, ON286951.1, ON152534.1, ON152533.1, ON152532.1, ON152531.1, ON152530.1, ON152529.1, ON152528.1, ON152527.1, ON152526.1, ON152525.1, ON152524.1, ON152523.1, ON152522.1, ON152521.1, ON152520.1, ON152519.1, ON152518.1, ON152517.1, ON152516.1, ON152515.1, ON152514.1, ON152513.1, ON152512.1, ON152511.1, ON152510.1, ON152509.1, ON152508.1, ON152507.1, ON152506.1, ON152505.1, ON152504.1, ON152503.1, ON152502.1, ON152501.1, ON152500.1, ON152499.1, ON152498.1, ON152497.1, ON152496.1, ON152495.1, ON152494.1, ON152493.1, ON152492.1, ON152491.1, ON152490.1, ON152489.1, ON152488.1, ON152487.1, ON152486.1, ON152485.1, ON152484.1, ON152483.1, ON152482.1, ON152481.1, ON152480.1, ON152479.1, ON152478.1, ON152477.1, ON152476.1, ON152475.1, ON152474.1, ON152473.1, ON152472.1, ON152471.1, ON152470.1, ON152469.1, ON152468.1, ON152467.1, ON152466.1, ON152465.1, ON152464.1, ON152463.1, ON152462.1, ON152461.1, ON152460.1, ON152459.1, ON152458.1, ON152457.1, ON152456.1, ON152455.1, ON152454.1, ON152453.1, ON152452.1, ON152451.1, ON152450.1, ON152449.1, ON152448.1, ON152447.1, ON152446.1, ON152445.1, ON152444.1, ON152443.1, ON152442.1, ON152441.1, ON152440.1, ON152439.1, ON152438.1, ON152437.1, ON152436.1, ON152435.1, ON152434.1, ON152433.1, ON152432.1, ON152431.1, ON152430.1, ON152429.1, ON152428.1, ON152427.1, ON152426.1, ON152425.1, ON152424.1, OL790292.1, OL790291.1, OL790290.1, OL790289.1, OL790288.1, OL790287.1, OL790286.1, OL790285.1, OL790284.1, OL790283.1, OL790282.1, OL790281.1, OL790280.1, OL790279.1, OL790278.1, OL790277.1, OL790276.1, OL790275.1, OL790274.1, OL790273.1, OL790272.1, OL790271.1, OL790270.1, OL790269.1, OL790268.1, OL790267.1, OL790266.1, OL790265.1, OL790264.1, OL790263.1, ON715115.1, ON715114.1, ON715113.1, ON715112.1, ON715111.1, ON715110.1, ON715109.1, ON715108.1, ON715107.1, ON715106.1, ON715105.1, ON715104.1, ON715103.1, ON715102.1, ON715101.1, ON715100.1, ON715099.1, ON715098.1, ON715097.1, ON715096.1, ON715095.1, ON715094.1, ON715093.1, ON715092.1, ON715091.1, ON715090.1, ON715089.1, ON715088.1, ON715087.1, ON715086.1, ON715085.1, ON715084.1, ON715083.1, ON715082.1, ON715081.1, ON715080.1, ON715079.1, ON715078.1, ON715077.1, ON715076.1, ON715075.1, ON715074.1, ON715073.1, ON715072.1, ON715071.1, ON715070.1, ON715069.1, ON715068.1, AY065636.1, AY065634.1, AY065633.1, MN832712.1, MN369438.1, MN369437.1, MN369436.1, MN369435.1, MN369434.1, MN369433.1, MN369432.1, MN369431.1, MN369430.1, MN369429.1, MN369428.1, MN369427.1, MN369426.1, MN369425.1, MN369424.1, MN369423.1, MN369422.1, MN369421.1, MN369420.1, MN369419.1, MN369418.1, MN369417.1, MN369416.1, MN369415.1, MN369414.1, MN369413.1, MN369412.1, MN369411.1, MN369410.1, MN369409.1, MN369408.1, MN369407.1, MN369406.1, MN369405.1, MN369404.1, MN369403.1, MN369402.1, MN369401.1, MN369400.1, MN369399.1, MN369398.1, MN369397.1, MN369396.1, MN369395.1, MN369394.1, MN369393.1, MN369392.1, MN369391.1, MN369390.1, MN369389.1, MN369388.1, MN369387.1, MN369386.1, MN369385.1, MN369384.1, MN369383.1, MN369382.1, MN369381.1, MN369380.1, MN369379.1, MN369378.1, MN369377.1, MN369376.1, MN369375.1, MN369374.1, MN369373.1, MN369372.1, MN369371.1, MN369370.1, MN369369.1, MN369368.1, MN369367.1, MN369366.1, MN369365.1, MN369364.1, MN369363.1, MN369362.1, MN369361.1, MN369360.1, MN369359.1, MN369358.1, MN369357.1, MN369356.1, MN369355.1, MN369354.1, MN369353.1, MN369352.1, MN369351.1, MN369350.1, MN369349.1, MN369348.1, MN369347.1, MN369346.1, MN369345.1, MN369344.1, MN369343.1, MN369342.1, MN369341.1, MN369340.1, MN369339.1, MN369338.1, MN369337.1, MN369336.1, MN369335.1, MN369334.1, MN369333.1, MN369332.1, MN369331.1, MN369330.1, MN369329.1, MN369328.1, MN369327.1, MN369326.1, MN369325.1, MN369324.1, MN369323.1, MN369322.1, MN369321.1, MN369320.1, MN369319.1, MN369318.1, MN369317.1, MN369316.1, MN369315.1, MN369314.1, MN369313.1, MN369312.1, MN369311.1, MN369310.1, MK798161.1, MK798160.1, MK798159.1, MG190868.1, MG190867.1, MG190866.1, MG190865.1, MG190864.1, MG190863.1, MG190862.1, MG190861.1, MG190860.1, LC319709.1, LC319708.1, LC319707.1, LC319706.1, LC319705.1, LC319704.1, LC319703.1, LC319702.1, LC319701.1, LC319700.1, LC319699.1, LC319698.1, LC319697.1, LC319696.1, LC319695.1, LC319694.1, LC319693.1, LC319692.1, LC319691.1, LC319690.1, LC319689.1, LC319688.1, LC319687.1, LC319686.1, LC319685.1, LC319684.1, LC319683.1, LC319682.1, LC319681.1, LC319680.1, LC319679.1, LC319678.1, LC319677.1, LC319676.1, LC319675.1, LC319674.1, LC319673.1, LC319672.1, KX759615.1, KX759614.1, KX759613.1, KX759612.1, KX759611.1, KX759610.1, KX759609.1, KX759608.1, KX759607.1, KX759606.1, KX759605.1, KX759604.1, KX759603.1, KX759602.1, KX759601.1, KX759600.1, KX759599.1, LC319674.1, LC319673.1, LC319672.1, KX759615.1, KX759614.1, KX759612.1, KX759611.1, KX759610.1, KX759609.1, KX759608.1, KX759607.1, KX759606.1, KX759605.1, KX759604.1, KX759603.1, KX759602.1, KX759601.1, KX759600.1, KX759599.1, KX759598.1, KX759597.1, KX759596.1, KX759595.1, KX759594.1, KX759593.1, KX759592.1, KX759591.1, KX759590.1, KX759589.1, KX759588.1, KX759587.1, KX759586.1, KX759585.1, KX759584.1, KX759583.1, KX759582.1, KX759581.1, KX759580.1, KX759579.1, KX759578.1, KX759577.1, KX759576.1, KX759575.1, KX759574.1, KX759573.1, KX759572.1, KX759571.1, KX759570.1, KX242298.1, EU835886.1, EU835885.1, EU835884.1, EU835883.1, EU835882.1, EU835881.1, EU835880.1, EU835879.1, EU835878.1, EU835877.1, EU835876.1, EU835875.1, EU835874.1, EU835873.1, EU835872.1, EU835871.1, EU835870.1, EU835869.1, EU835868.1, EU835867.1, EU835866.1, EU835865.1, EU835864.1, EU835863.1, EU835862.1, EU835861.1, EU835860.1, EU835859.1, DQ297826.1, DQ297825.1, DQ297824.1, DQ297823.1, DQ297822.1, DQ297821.1, AY665670.1, AY665669.1, AY665668.1, AY100319.1, AY100321.1, AY100320.1, AY100000.1, AY099999.1, AY099997.1, AY099998.1, AY095534.1, AY095229.1, AY095230.1, AY095231.1, AF498635.1, AF498634.1, AF498633.1, AF498632.1, AF498631.1, AF498630.1, AF498629.1, AF498628.1, AF498623.1, AF498622.1, AF498620.1, AF498619.1, AF498618.1, AY305387.1, AY305386.1, AF091099.1, AF091098.1, AF091097.1, FJ607182.1, FJ607181.1, FJ607180.1, FJ607179.1, FJ607178.1, FJ607177.1, FJ607176.1, FJ607175.1, FJ607174.1, FJ607173.1, FJ607172.1, FJ607171.1, FJ607170.1, FJ607169.1, FJ607168.1, FJ607167.1, FJ607166.1, AY012683.1, AY012682.1, AY012681.1, AY012680.1, AY012679.1, AY012678.1, AY012677.1, AF304028.1, AF304027.1, AF304026.1, AF304025.1, KP266334.1, KP266333.1, KP266332.1, KP266331.1, KP266330.1, KP266329.1, KP266328.1, KP266327.1, KP266326.1, KP266325.1, KP266324.1, KP266323.1, KP266322.1, KP152322.1, KP152321.1, KP152320.1, KP152319.1, KP152318.1, KP152317.1, KP152316.1, KP152315.1, KP152314.1, KP152313.1, KP152312.1, KP152311.1, KP152310.1, KP152309.1, KP152308.1, KP152307.1, KP152306.1, KP152305.1, KP152304.1, KP152303.1, KP152302.1, KP152301.1, KP152300.1, KP152299.1, KP152298.1, KP152297.1, KP152296.1, KP152295.1, KP152294.1, KP152293.1, KP152292.1, KP152291.1, KP152290.1, KP152289.1, KP152288.1, KP152287.1, KP152286.1, KP152285.1, KP152284.1, KP152283.1, KP152282.1, KP152281.1, KP152280.1, KP152279.1, KP152278.1, KP152277.1, KP152276.1, KP152275.1, KP152274.1, KP152273.1, KP152272.1, KP152271.1, KP152270.1, KP152269.1, KP152268.1, KP152267.1, KP152266.1, KP152265.1, KP152264.1, KP152263.1, KP152262.1, KP152261.1, KP152260.1, KP152259.1, KP152258.1, KP152257.1, KP152256.1, KP152255.1, KP152254.1, KP152253.1, KP152252.1, KP152251.1, KP152250.1, KP152249.1, KP152248.1, KP152247.1, KP152246.1, KP152245.1, KP152244.1, KP152243.1, KP152242.1, KP152241.1, KP152240.1, KP152239.1, KP152238.1, KP152237.1, KP152236.1, KP152235.1, KP152234.1, KP152233.1, KP152232.1, KP152231.1, KP864049.1, KP864048.1, KP864047.1, KP864046.1, JX974583.1, JX974582.1, AY770593.1, AY770592.1, AY770591.1, AY770590.1, AY770589.1, AY770588.1, AY770587.1, AY770586.1, AY770585.1, AY770584.1, AY770583.1, AY770582.1, AY770581.1, AY525119.1, AY525118.1, AY525117.1, AY525116.1, AY525115.1, AY525114.1, AY525113.1, AY525112.1, AY525111.1, AY525110.1, AY525109.1, AY525108.1, AY525107.1, JN712821.1, JN712820.1, JN712819.1, JN712818.1, JN712817.1, JN712816.1, JN712815.1, JN712814.1, JN712813.1, JN712812.1, EU224390.1, FM205023.1, AJ632141.1, AJ878913.1, AJ878912.1, AJ878911.1, AJ878910.1, AJ878909.1, AJ878908.1, AJ878907.1, AJ878906.1, AJ878905.1, AJ878904.1, AJ878903.1, AJ878902.1, AJ878901.1, AJ878900.1, AJ878899.1, AJ878898.1, AJ878897.1, AJ878896.1, AJ878895.1, AJ878894.1, AJ878893.1, AJ878892.1, AJ878891.1, AJ878890.1, AJ878889.1, AJ878888.1, AJ878887.1, AJ878886.1, AJ878885.1, AJ878884.1, AJ878883.1, AJ878882.1, AJ583500.1, AJ872115.1, AY359864.1, AY359863.1, OQ756336.1, MZ665486.1, MN917660.1, MN917659.1, MN917658.1, MN917657.1, MN917656.1, MN917655.1, EU025733.1, EU025732.1, EU025731.1, EU025730.1, EU025729.1, EU025728.1, EU025727.1, EU025726.1, EU025725.1, EU025724.1, EU025723.1, EU025722.1, EU025721.1, EU025720.1, MN052569.1, MN052568.1, MN052567.1, MN052566.1, MN052565.1, MN052564.1, MN052563.1, MN052562.1, MN052561.1, MN052560.1, MN241439.1, MN241438.1, MN241437.1, MN241436.1, MN241435.1, MN241434.1, MN241433.1, MK333523.1, MK333522.1, MK333521.1, MK333520.1, MK333519.1, MK318945.1, KY411647.1, KY411646.1, KY411645.1, KY411644.1, KY411643.1, KY411642.1, AY065636.1, AY065634.1, AY065633.1, MN832712.1, MN369438.1, MN369437.1, MN369436.1, MN369435.1, MN369434.1, MN369433.1, MN369432.1, MN369431.1, MN369430.1, MN369429.1, MN369428.1, MN369427.1, MN369426.1, MN369425.1, MN369424.1, MN369423.1, MN369422.1, MN369421.1, MN369420.1, MN369419.1, MN369418.1, MN369417.1, MN369416.1, MN369415.1, MN369414.1, MN369413.1, MN369412.1, MN369411.1, MN369410.1, MN369409.1, MN369408.1, MN369407.1, MN369406.1, MN369405.1, MN369404.1, MN369403.1, MN369402.1, MN369401.1, MN369400.1, MN369399.1, MN369398.1, MN369397.1, MN369396.1, MN369395.1, MN369394.1, MN369393.1, MN369392.1, MN369391.1, MN369390.1, MN369389.1, MN369388.1, MN369387.1, MN369386.1, MN369385.1, MN369384.1, MN369383.1, MN369382.1, MN369381.1, MN369380.1, MN369379.1, MN369378.1, MN369377.1, MN369376.1, MN369375.1, MN369374.1, MN369373.1, MN369372.1, MN369371.1, MN369370.1, MN369369.1, MN369368.1, MN369367.1, MN369366.1, MN369365.1, MN369364.1, MN369363.1, MN369362.1, MN369361.1, MN369360.1, MN369359.1, MN369358.1, MN369357.1, MN369356.1, MN369355.1, MN369354.1, MN369353.1, MN369352.1, MN369351.1, MN369350.1, MN369349.1, MN369348.1, MN369347.1, MN369346.1, MN369345.1, MN369344.1, MN369343.1, MN369342.1, MN369341.1, MN369340.1, MN369339.1, MN369338.1, MN369337.1, MN369336.1, MN369335.1, MN369334.1, MN369333.1, MN369332.1, MN369331.1, MN369330.1, MN369329.1, MN369328.1, MN369327.1, MN369326.1, MN369325.1, MN369324.1, MN369323.1, MN369322.1, MN369321.1, MN369320.1, MN369319.1, MN369318.1, MN369317.1, MN369316.1, MN369315.1, MN369314.1, MN369313.1, MN369312.1, MN369311.1, MN369310.1, MK798161.1, MK798160.1, MK798159.1, MG190868.1, MG190867.1, MG190866.1, MG190865.1, MG190864.1, MG190863.1, MG190862.1, MG190861.1, MG190860.1, LC319709.1, LC319708.1, LC319707.1, LC319706.1, LC319705.1, LC319704.1, LC319703.1, LC319702.1, LC319701.1, LC319700.1, LC319699.1, LC319698.1, LC319697.1, LC319696.1, LC319695.1, LC319694.1, LC319693.1, LC319692.1, LC319691.1, LC319690.1, LC319689.1, LC319688.1, LC319687.1, LC319686.1, LC319685.1, LC319684.1, LC319683.1, LC319682.1, LC319681.1, LC319680.1, LC319679.1, LC319678.1, LC319677.1, LC319676.1, LC319675.1, LC319674.1, LC319673.1, LC319672.1, KX759615.1, KX759614.1, KX759613.1, KX759612.1, KX759611.1, KX759610.1, KX759609.1, KX759608.1, KX759607.1, KX759606.1, KX759605.1, KX759604.1, KX759603.1, KX759602.1, KX759601.1, KX759600.1, KX759599.1, LC319674.1, LC319673.1, LC319672.1, KX759615.1, KX759614.1, KX759612.1, KX759611.1, KX759610.1, KX759609.1, KX759608.1, KX759607.1, KX759606.1, KX759605.1, KX759604.1, KX759603.1, KX759602.1, KX759601.1, KX759600.1, KX759599.1, KX759598.1, KX759597.1, KX759596.1, KX759595.1, KX759594.1, KX759593.1, KX759592.1, KX759591.1, KX759590.1, KX759589.1, KX759588.1, KX759587.1, KX759586.1, KX759585.1, KX759584.1, KX759583.1, KX759582.1, KX759581.1, KX759580.1, KX759579.1, KX759578.1, KX759577.1, KX759576.1, KX759575.1, KX759574.1, KX759573.1, KX759572.1, KX759571.1, KX759570.1, KX242298.1, EU835886.1, EU835885.1, EU835884.1, EU835883.1, EU835882.1, EU835881.1, EU835880.1, EU835879.1, EU835878.1, EU835877.1, EU835876.1, EU835875.1, EU835874.1, EU835873.1, EU835872.1, EU835871.1, EU835870.1, EU835869.1, EU835868.1, EU835867.1, EU835866.1, EU835865.1, EU835864.1, EU835863.1, EU835862.1, EU835861.1, EU835860.1, EU835859.1, DQ297826.1, DQ297825.1, DQ297824.1, DQ297823.1, DQ297822.1, DQ297821.1, AY665670.1, AY665669.1, AY665668.1, AY100319.1, AY100321.1, AY100320.1, AY100000.1, AY099999.1, AY099997.1, AY099998.1, AY095534.1, AY095229.1, AY095230.1, AY095231.1, AF498635.1, AF498634.1, AF498633.1, AF498632.1, AF498631.1, AF498630.1, AF498629.1, AF498628.1, AF498623.1, AF498622.1, AF498620.1, AF498619.1, AF498618.1, AY305387.1, AY305386.1, AF091099.1, AF091098.1, AF091097.1, FJ607182.1, FJ607181.1, FJ607180.1, FJ607179.1, FJ607178.1, FJ607177.1, FJ607176.1, FJ607175.1, FJ607174.1, FJ607173.1, FJ607172.1, FJ607171.1, FJ607170.1, FJ607169.1, FJ607168.1, FJ607167.1, FJ607166.1, AY012683.1, AY012682.1, AY012681.1, AY012680.1, AY012679.1, AY012678.1, AY012677.1, AF304028.1, AF304027.1, AF304026.1, AF304025.1, KP266334.1, KP266333.1, KP266332.1, KP266331.1, KP266330.1, KP266329.1, KP266328.1, KP266327.1, KP266326.1, KP266325.1, KP266324.1, KP266323.1, KP266322.1, KP152322.1, KP152321.1, KP152320.1, KP152319.1, KP152318.1, KP152317.1, KP152316.1, KP152315.1, KP152314.1, KP152313.1, KP152312.1, KP152311.1, KP152310.1, KP152309.1, KP152308.1, KP152307.1, KP152306.1, KP152305.1, KP152304.1, KP152303.1, KP152302.1, KP152301.1, KP152300.1, KP152299.1, KP152298.1, KP152297.1, KP152296.1, KP152295.1, KP152294.1, KP152293.1, KP152292.1, KP152291.1, KP152290.1, KP152289.1, KP152288.1, KP152287.1, KP152286.1, KP152285.1, KP152284.1, KP152283.1, KP152282.1, KP152281.1, KP152280.1, KP152279.1, KP152278.1, KP152277.1, KP152276.1, KP152275.1, KP152274.1, KP152273.1, KP152272.1, KP152271.1, KP152270.1, KP152269.1, KP152268.1, KP152267.1, KP152266.1, KP152265.1, KP152264.1, KP152263.1, KP152262.1, KP152261.1, KP152260.1, KP152259.1, KP152258.1, KP152257.1, KP152256.1, KP152255.1, KP152254.1, KP152253.1, KP152252.1, KP152251.1, KP152250.1, KP152249.1, KP152248.1, KP152247.1, KP152246.1, KP152245.1, KP152244.1, KP152243.1, KP152242.1, KP152241.1, KP152240.1, KP152239.1, KP152238.1, KP152237.1, KP152236.1, KP152235.1, KP152234.1, KP152233.1, KP152232.1, KP152231.1, KP864049.1, KP864048.1, KP864047.1, KP864046.1, JX974583.1, JX974582.1, AY770593.1, AY770592.1, AY770591.1, AY770590.1, AY770589.1, AY770588.1, AY770587.1, AY770586.1, AY770585.1, AY770584.1, AY770583.1, AY770582.1, AY770581.1, AY525119.1, AY525118.1, AY525117.1, AY525116.1, AY525115.1, AY525114.1, AY525113.1, AY525112.1, AY525111.1, AY525110.1, AY525109.1, AY525108.1, AY525107.1, JN712821.1, JN712820.1, JN712819.1, JN712818.1, JN712817.1, JN712816.1, JN712815.1, JN712814.1, JN712813.1, JN712812.1, EU224390.1, FM205023.1, AJ632141.1, AJ878913.1, AJ878912.1, AJ878911.1, AJ878910.1, AJ878909.1, AJ878908.1, AJ878907.1, AJ878906.1, AJ878905.1, AJ878904.1, AJ878903.1, AJ878902.1, AJ878901.1, AJ878900.1, AJ878899.1, AJ878898.1, AJ878897.1, AJ878896.1, AJ878895.1, AJ878894.1, AJ878893.1, AJ878892.1, AJ878891.1, AJ878890.1, AJ878889.1, AJ878888.1, AJ878887.1, AJ878886.1, AJ878885.1, AJ878884.1, AJ878883.1, AJ878882.1, AJ583500.1, AJ872115.1, AY359864.1, AY359863.1, OQ756336.1, MZ665486.1, MN917660.1, MN917659.1, MN917658.1, MN917657.1, MN917656.1, MN917655.1, EU025733.1, EU025732.1, EU025731.1, EU025730.1, EU025729.1, EU025728.1, EU025727.1, EU025726.1, EU025725.1, EU025724.1, EU025723.1, EU025722.1, EU025721.1, EU025720.1, MN052569.1, MN052568.1, MN052567.1, MN052566.1, MN052565.1, MN052564.1, MN052563.1, MN052562.1, MN052561.1, MN052560.1, MN241439.1, MN241438.1, MN241437.1, MN241436.1, MN241435.1, MN241434.1, MN241433.1, MK333523.1, MK333522.1, MK333521.1, MK333520.1, MK333519.1, MK318945.1, KY411647.1, KY411646.1, KY411645.1, KY411644.1, KY411643.1, KY411642.1, EF139035.1, EF139034.1, EF139033.1, EF139032.1, EF139031.1, EF139030.1, EF139029.1, EF139028.1, EF139027.1, EF139026.1, EF139025.1, EF139024.1, EF139023.1, EF139022.1, EF139021.1, EF139020.1, EF139019.1, EF139018.1, EF139017.1, EF139016.1, EF139015.1, EF139014.1, EF139013.1, EF139012.1, EF139011.1, EF139010.1, EF139009.1, EF139008.1, EF139007.1, EF139006.1, EF139005.1, EF139004.1, EF139003.1, EF139002.1, EF139001.1, EF139000.1, EF138999.1, EF138998.1, EF138997.1, EF138996.1, EF138995.1, EF138994.1, EF138993.1, EF138992.1, EF138991.1, EF138990.1, EF138989.1, EF138988.1, EF138987.1, EF138986.1, EF138985.1, EF138984.1, EF138983.1, EF138982.1, EF138981.1, EF138980.1, EF138979.1, EF138978.1, EF138977.1, EF138976.1, EF138975.1, EF138974.1, EF138973.1, EF138972.1, EF138971.1, EF138970.1, EF138969.1, EF138968.1, EF138967.1, EF138966.1, EF138965.1, EF138964.1, EF138963.1, EF138962.1, EF138961.1, EF138960.1, EF138959.1, EF138958.1, EF138957.1, EF138956.1, EF138955.1, EF138954.1, EF138953.1, EF138952.1, EF138951.1, EF138950.1, EF138949.1, EF138948.1, DQ899752.2, DQ899754.1, DQ899753.1, DQ899751.1, DQ899750.1, DQ659246.1, DQ656522.1, DQ656521.1, DQ656520.1, DQ656519.1, DQ656518.1, DQ656517.1, DQ656516.1, DQ656515.1, DQ656514.1, DQ656513.1, DQ656512.1, DQ656511.1, DQ656507.1, DQ656506.1, DQ656505.1, DQ656504.1, DQ656503.1, DQ656502.1, DQ656501.1, DQ656500.1, DQ656499.1, DQ656498.1, DQ656497.1, DQ656496.1, DQ656495.1, DQ656494.1, DQ630460.1, DQ630459.1, DQ630458.1, DQ630457.1, DQ630456.1, DQ630455.1, DQ630454.1, DQ630453.1, DQ630452.1, DQ630451.1, DQ630450.1, DQ630449.1, DQ630448.1, DQ630447.1, DQ630446.1, DQ630445.1, KX017539.1, KX017538.1, KU994832.1, KU994831.1, KT630856.1, KT630855.1, KT630854.1, KT630853.1, KT630852.1, KT630851.1, KT630850.1, KT630849.1, KU302608.1, KU302607.1, KU302606.1, KP878300.1, KM523661.1, KM523660.1, KM523659.1, KM523658.1, KM523657.1, KM523656.1, KM523655.1, KM523654.1, KM523653.1, KM523652.1, KM523651.1, KM523650.1, KM523649.1, KM523648.1, KM523647.1, KM523646.1, KM523645.1, KM523644.1, KM523643.1, KM523642.1, KM523641.1, KM523640.1, KM523639.1, KM523638.1, KM523637.1, KM523636.1, KM523635.1, KM523634.1, KM523633.1, KM523632.1, KM523631.1, KM523630.1, KM523629.1, KM523628.1, KM523627.1, KR028197.1, KR028196.1, KR028195.1, KF013951.1, KM012167.1, KM012166.1, KM012165.1, KM012164.1, KM012163.1, KM012162.1, KM012161.1, KM012160.1, KM012159.1, KM012158.1, KM012157.1, KM012156.1, KM012155.1, KM012154.1, KM012153.1, KM012152.1, KM012151.1, KM012150.1, KM012149.1, KM012148.1, KM012147.1, KM012146.1, KM012145.1, KM012144.1, KM012143.1, KM012142.1, KM012141.1, KM012140.1, KM012139.1, KM012138.1, KM012137.1, KM012136.1, KM012135.1, KM012134.1, KM012133.1, KM012132.1, KM012131.1, KM012130.1, KM012129.1, KM012128.1, KM012127.1, KM012126.1, KM012125.1, KM012124.1, KM012123.1, KM012122.1, KM012121.1, KM012120.1, KM012119.1, KM012118.1, KM012117.1, KM012116.1, KM012115.1, KM887851.1, KM887850.1, KM887849.1, KM887848.1, KM887847.1, KM887846.1, KM979219.1, KM979218.1, KM979217.1, KM979216.1, KM979215.1, KJ621065.1, KJ621064.1, KJ621063.1, KJ621062.1, KJ621061.1, KJ621060.1, KJ621059.1, KJ621058.1, KJ621057.1, KJ621056.1, KJ621055.1, KJ621054.1, KJ621053.1, KJ621052.1, KJ621051.1, KJ621050.1, KF439863.1, JF682320.1, JF682319.1, JF682318.1, JF682317.1, JF682316.1, JF682315.1, JF682314.1, JF682313.1, JF682312.1, JF682311.1, JF682310.1, JF682309.1, JF682308.1, JF682307.1, JF682306.1, JF682305.1, JF682304.1, JF682303.1, JF682302.1, JF682301.1, JF682300.1, JF682299.1, JF682298.1, JF682297.1, JF682296.1, JF682295.1, JF682294.1, JF682293.1, JF682292.1, JF682291.1, JF682290.1, JF682289.1, JF682288.1, JF682287.1, JF682286.1, JF682285.1, JF682284.1, JF682283.1, JF682282.1, JF682281.1, JF682280.1, JF682279.1, JF682278.1, JF682277.1, JF682276.1, JF682275.1, JF682274.1, JF682273.1, JF682272.1, JF682271.1, JF682270.1, JF682269.1, JF682268.1, JF682267.1, JF682266.1, JF682265.1, JF682264.1, JF682263.1, JF682262.1, JF682261.1, JF682260.1, JF682259.1, JF682258.1, JF682257.1, JF682256.1, JF682255.1, JF682254.1, JF682253.1, JF682252.1, JF682251.1, JF682250.1, JF682249.1, JF682248.1, JF682247.1, JF682246.1, JF682245.1, JF682244.1, JQ286018.1, JQ286017.1, JQ353518.1, JQ353517.1, JQ353516.1, JQ353515.1, JQ353514.1, JQ353513.1, JQ353512.1, JQ353511.1, JQ353510.1, JQ353509.1, JQ353508.1, JQ353507.1, JQ353506.1, JQ353505.1, JQ353504.1, JQ353503.1, JQ353502.1, JQ353501.1, JQ353500.1, JQ353499.1, JQ353498.1, JQ353497.1, JQ353496.1, JQ353495.1, JQ353494.1, JQ353493.1, JQ353492.1, JQ353491.1, JQ353490.1, JQ353489.1, JQ001867.1, PP492702.1, PP492701.1, PP492700.1, PP492699.1, PP492698.1, PP492697.1, PP492696.1, PP492695.1, PP492694.1, PP492693.1, PP273510.1, OM056536.1, OM056535.1, OL803871.1, OL696045.1, OL696044.1, OL696043.1, OL696042.1, OL696041.1, OR611976.1, OR611975.1, OR418342.1, OR418341.1, OR418340.1, OR168974.1, OR166261.1, OQ534291.1, OQ534290.1, OQ427406.1, OQ427405.1, LC760551.1, LC760550.1, LC760549.1, LC760548.1, LC760547.1, LC760546.1, LC760545.1, LC760544.1, LC760543.1, ON567384.1, OM908471.1, OM908470.1, OM908469.1, OM908468.1, OM908467.1, OM908466.1, OM908465.1, OM908464.1, OM908463.1, OM908462.1, OM908461.1, OM908460.1, OM908459.1, OM908458.1, OM908457.1, OM908456.1, OM908455.1, OM908454.1, OM908453.1, OM908452.1, OM908451.1, OM908450.1, OM908449.1, OM908448.1, OM908447.1, OM908446.1, OM908445.1, OM908444.1, OM908443.1, OM908442.1, OM908441.1, OM908440.1, OM908439.1, OM851051.1, OM851050.1, OM851049.1, OM851048.1, OM851047.1, OM851046.1, OM851045.1, OM851044.1, OM851043.1, OM851042.1, OM851041.1, OM851040.1, OM851039.1, OM851038.1, OM851037.1, OM851036.1, ON792311.1, ON792310.1, ON792309.1, OM202462.1, MZ044949.1, MZ044948.1, MZ044947.1, MZ044946.1, MZ044945.1, MZ044944.1, OM791285.1, OM791284.1, OM791283.1, OM791282.1, OM791281.1, OM791280.1, OM791279.1, OM791278.1, OM791277.1, OM791276.1, OM791275.1, OM791274.1, OM791273.1, OM791272.1, OM791271.1, OK483322.1, MW367236.1, MW367235.1, MW367234.1, MW367233.1, MW367232.1, MW367231.1, MW367230.1, MW367229.1, MW367228.1, MW367227.1, MW075221.1, OL690423.1, OL690422.1, OL690421.1, MT992253.1, MT992252.1, MT992251.1, MT992250.1, MT992249.1, MT992248.1, MT992247.1, MT992246.1, MT992245.1, MT992244.1, MZ409478.1, MW316418.1, MW316417.1, MT090079.1, MT090078.1, MN823616.1, MT642698.1, MT642697.1, MT642696.1, MT642695.1, MT642694.1, MT642693.1, MT642692.1, MT642691.1, MT642690.1, MT642689.1, MT642688.1, MT642687.1, MT642686.1, MT642685.1, MT642684.1, MT642683.1, MT642682.1, MT642681.1, MT642680.1, MT642679.1, MT642678.1, MT642677.1, MT642676.1, MT642675.1, MT642674.1, MT642673.1, MT642672.1, MT642671.1, MT642670.1, MN912479.1, MN912478.1, MN912477.1, MN912476.1, MN912475.1, MN912474.1, MN906755.1, MN900865.1, MT104576.1, MT104575.1, MT050443.1, MT050442.1, MT050441.1, MT050440.1, MT050439.1, MT050438.1, MT050437.1, MT050436.1, MT050435.1, EU328335.1, EU328334.1, EU328333.1, EU328332.1, OR206424.1, OR206423.1, KY450715.1, KY450714.1, KY450713.1, KY450712.1, KY407655.1, KY407654.1, KY407653.1, KY407652.1, KY407651.1, KY407650.1, KY407649.1, KY407648.1, KY407647.1, KY407646.1, KY407645.1, KY407644.1, KY407643.1, KY407642.1, KY407641.1, KY407640.1, KY407639.1, KY407638.1, KY407637.1, KY407636.1, KY407635.1, KY407634.1, KY407633.1, KY407632.1, KY407631.1, KY407630.1, KY407629.1, KY407628.1, KY407627.1, KY407626.1, KY407625.1, KY407624.1, KY407623.1, KY407622.1, KY407621.1, KY407620.1, KY407619.1, KY407618.1, KY407617.1, KY407616.1, KY407615.1, KY407614.1, KY407613.1, KY407612.1, KY407611.1, KY407610.1, KY407609.1, KY407608.1, KY407607.1, KY407606.1, KY407605.1, KY407604.1, KY407603.1, KY407602.1, KY407601.1, KY407600.1, KY407599.1, KY407598.1, KY407597.1, KY407596.1, KY407595.1, KY407594.1, KY407593.1, KY407592.1, KY407591.1, KY407590.1, KY407589.1, KY407588.1, KY407587.1, KY407586.1, KY407585.1, KY407584.1, KY407583.1, KY407582.1, KY407581.1, MF113489.1, MF113488.1, KY000833.1, DQ000436.1, AY737506.1, AY737505.1, AY737504.1, AY737503.1, AY737502.1, AY737501.1, AY737500.1, AY737499.1, AY737498.1, AY780348.1, AY362979.1, AY362978.1, AY333088.1, AY367560.1, AY311479.1, FJ824699.1, EF066501.1, EF066500.1, EF066499.1, EF066498.1, EF066497.1, EF066496.1, EF066495.1, EF066494.1, EF066493.1, EF066492.1, EF066491.1, AF312371.1, KX011053.1, KX011052.1, KX011051.1, KX011050.1, KX011049.1, KX011048.1, KX011047.1, KX011046.1, KX011045.1, KX011044.1, KU058689.1, KU058688.1, KU058687.1, KU058686.1, KU058685.1, KM870814.1, KM870813.1, KM870812.1, KM870811.1, KM870810.1, KM870809.1, KM870808.1, KM659895.1, KM659894.1, KM659893.1, KM659892.1, KM659891.1, KM659890.1, KM659889.1, KM659888.1, KM659887.1, KM659886.1, KM659885.1, KM659884.1, KM659883.1, KM659882.1, KM659881.1, JQ315183.1, JQ315162.1, JQ315161.1, JQ315160.1, JQ315159.1, JQ315158.1, GQ131546.1, GQ131545.1, GQ131544.1, GQ131543.1, GQ131542.1, GQ131541.1, GQ131540.1, AY791998.1, Z97013.1 , Z97011.1 , Z97009.1 , Z97008.1 , Z97007.1 , Z97006.1 , Z97005.1 , Z97004.1 , Z97002.1 , Z97001.1 , Z97000.1 , Z96999.1 , Z96998.1 , Z96997.1 , Z96996.1 , PP353662.1, PP353661.1, PP353660.1, PP353659.1, PP353658.1, PP353657.1, PP353656.1, PP353655.1, PP353654.1, PP353653.1, PP353652.1, PP353651.1, PP353650.1, OQ929506.1, MZ041716.1, MW483684.1, MW483683.1, MW483682.1, MW483681.1, MW297135.1, MN737291.1, MN737290.1, MN737289.1, MN737288.1, MN737287.1, MN737286.1, MN737285.1, MN737284.1, MN737283.1, MN737282.1, MN737281.1, MN737280.1, MN737279.1, MN737278.1, MN737277.1, MN737276.1, MN737275.1, MN737274.1, MN737273.1, MN737272.1, MN737271.1, MN737270.1, MK803311.1, MK803310.1, MK803309.1, MK803308.1, MK803307.1, MK803306.1, MK803305.1, MK803304.1, MK803303.1, MK803302.1, MK803301.1, MK803300.1, MK803299.1, MK803298.1, MK803297.1, MK803296.1, MK803295.1, MK803294.1, MK803293.1, MK803292.1, MK803291.1, MK803290.1, MK803289.1, MK803288.1, MK803287.1, MK803286.1, MK803285.1, MK803284.1, MK803283.1, MN096599.1, MK507834.1, MF043591.1, MF043590.1, DQ324535.1, DQ324534.1, DQ324533.1, DQ324532.1, DQ324531.1, AY780347.1, AY780346.1, AY780345.1, AY780344.1, AY780343.1, AY780342.1, AY780341.1, AY780340.1, AY780339.1, AY780338.1, AY780337.1, AY780336.1, AY780335.1, AY780334.1, AY780333.1, AY780332.1, AY780331.1, AY780330.1, AY780329.1, AY780328.1, AY780327.1, AY780326.1, AY780325.1, AY780324.1, AY780323.1, AY780322.1, AY780321.1, AY780320.1, AY780319.1, AY780318.1, AY780317.1, AY780316.1, AY780315.1, AY780314.1, AY780313.1, AY780312.1, AY780311.1, AY780310.1, AY359862.1, AY318758.1, KX077979.1, KX077978.1, KX077977.1, KX077976.1, LC080693.1, LC080692.1, LC080691.1, LC080690.1, LC080689.1, LC080688.1, LC080687.1, LC080686.1, LC080685.1, LC080684.1, LC080683.1, LC080682.1, LC080681.1, LC080680.1, LC080679.1, LC080678.1, KT381974.1, LC001783.1, LC001782.1, LC001781.1, LC001780.1, LC001779.1, LC001778.1, LC001777.1, LC001776.1, LC001775.1, KM065458.1, JF736011.1, JF736010.1, JF736009.1, JF736008.1, JF736007.1, JF736006.1, JF736005.1, JF736004.1, JF736003.1, JF736002.1, JF736001.1, JF736000.1, JF735999.1, AB425829.1, AB425828.1, AB425827.1, AB425826.1, AB425825.1, AB425321.1, AJ508762.1, AJ508761.1, AJ508760.1, AJ508759.1, AJ508758.1, Y14963.1 , Y14962.1 , Y14961.1 , Y14960.1 , Y14959.1 , Y14958.1 , Y14957.1 , Y14956.1 , Y14955.1 , Z97014.1 , Z97012.1 , Z97010.1 , Z97003.1 , Z96995.1 , Z96994.1 , Z96993.1 , AJ315028.1, AJ315027.1, AJ315026.1, AJ404327.1, AJ277801.1, OQ929057.1, OQ929056.1, OQ929055.1, OQ929054.1, OQ929053.1, OQ929052.1, OQ929051.1, OQ929050.1, OQ929049.1, OQ929048.1, OQ929047.1, OQ929046.1, OQ929045.1, OQ929044.1, OQ929043.1, OQ929042.1, OQ929041.1, OQ929040.1, OQ929039.1, OQ929038.1, OQ929037.1, OQ929036.1, OQ929035.1, OQ929034.1, OQ929033.1, OQ929032.1, OQ929031.1, OQ929030.1, OQ929029.1, AF281240.1, AF281239.1, AF281238.1, AF281237.1, AF281236.1, AF281235.1, AF281234.1, AF281233.1, AF281232.1, AF281231.1, MK861041.1, MK861040.1, MK861039.1, MK861038.1, MK861037.1, MK861036.1, MK861035.1, MK861034.1, MK861033.1, MK861032.1, MK861031.1, MK861030.1, MK861029.1, MK861028.1, MK861027.1, MK861026.1, MK861025.1, MK861024.1, MK861023.1, MK861022.1, MK861021.1, MK861020.1, AY736545.1, AF498636.1, AF498627.1, AF498626.1, AF498625.1, AF498624.1, AF491865.1, AY083933.1, AY083932.1, AY083931.1, AY083930.1, AY083929.1, AY083928.1, AY083927.1, AY083926.1, AY083925.1, KT991840.1, KT991839.1, KT991838.1, KT991837.1, KT991836.1, JQ684022.1, JQ684021.1, JQ684020.1, JQ684019.1, JQ684018.1, JQ684017.1, JQ684016.1, JQ684015.1, JQ928404.1, JQ928403.1, JQ928402.1, JQ928401.1, JQ928400.1, JQ928399.1, JQ928398.1, JQ928397.1, JQ928396.1, JQ928395.1, JQ928394.1, JQ928393.1, JQ928392.1, JQ928391.1, JQ928390.1, JQ928389.1, JQ928388.1, JQ928387.1, JQ928386.1, JQ928385.1, JQ928384.1, JQ928383.1, JQ928382.1, JQ928381.1, JQ928380.1, JQ928379.1, JQ928378.1, JQ928377.1, JQ928376.1, JQ928375.1, JQ911703.1, JQ911702.1, AF006701.1, AF006700.1, AF006699.1, AF006698.1, AF006697.1, AF006696.1, AF006695.1, AF006694.1, X89570.1 , AJ416445.2, AJ416444.2, AJ504473.1, AJ295029.1, AJ001948.1, AJ001947.1, AJ001946.1, AJ001945.1, AJ001944.1, AJ001943.1, AJ001942.1, AJ001941.1, AJ249524.1, AJ249522.1, AJ249521.1, AJ249520.1, AJ249519.1, AJ249518.1, AJ245886.1, AJ245884.1, AJ245883.1, AJ249523.1, AJ249517.1, Y18612.1 , Y18682.1 , Y18650.1 , X96430.1 , X96718.1 , X96472.1 , PP098577.1, PP098576.1, PP098575.1, PP098574.1, PP098573.1, PP098572.1, PP098571.1, PP098570.1, PP098569.1, PP098568.1, OR900021.1, OR345746.1, OR345745.1, OR345744.1, OR345743.1, OR345742.1, OR345741.1, OR345740.1, MZ395297.1, MZ395296.1, MW451238.1, MW451237.1, MW451236.1, MW451235.1, MW451234.1, MW451233.1, MW451232.1, MW451231.1, MW451230.1, MW451229.1, MW451228.1, MW451227.1, MW451226.1, MT840281.1, MT840278.1, MT840277.1, MT840270.1, MT840268.1, MT840267.1, MT840264.1, MT840262.1, EU224392.1, EU224391.1, EU328331.1, EU328330.1, EU328329.1, EU328328.1, EU328327.1, EU328326.1, EU119865.1, EU086601.1, MN102364.1, MK906027.1, MK109006.1, MK109005.1, MK088026.1, MK088025.1, KY597857.1, KY597844.1, KY597843.1, KY597842.1, KY597841.1, KY597840.1, KY597839.1, KY597838.1, KY597837.1, KY597836.1, KY597835.1, KY597834.1, KY597833.1, MH078256.1, MH078255.1, MH078254.1, MH078253.1, MH137957.1, MH137956.1, MH137955.1, MH137954.1, MH137953.1, MH137952.1, MH137951.1, MH137950.1, MH137949.1, MH137948.1, MH137947.1, MH137946.1, MH137945.1, MH137944.1, MH137943.1, MH137942.1, KY973955.1, KY973954.1, KY973953.1, KY973952.1, MF320270.1, MF320269.1, MF320268.1, MF320267.1, MF320266.1, MF320265.1, MF320264.1, MF320263.1, MF320262.1, MF320261.1, MF320260.1, MF320259.1, MF320258.1, MF320257.1, KY523069.1, KY523068.1, KY523067.1, KY556581.1, KY556580.1, KY556579.1, KY556578.1, KY556577.1, KY556576.1, KY556575.1, KY556574.1, KY556573.1, KY556572.1, KY556571.1, KY556570.1, KY556569.1, KY556568.1, KY556567.1, KY556566.1, KY556565.1, KY556564.1, KY556563.1, KY556562.1, KY556561.1, KY556560.1, KY556559.1, KY556558.1, KY556557.1, KY556556.1, KY556555.1, KY556554.1, KY612983.1, KY612982.1, KY612981.1, KY612980.1, KY612979.1, KY612978.1, KY612977.1, KY612976.1, KY612975.1, KY612974.1, KY612973.1, KY612972.1, KY612971.1, KY612970.1, KY612969.1, KY612968.1, KY612967.1, KY612966.1, KY612965.1, KY612964.1, KY612963.1, KY612962.1, KY612961.1, KY612960.1, KY612959.1, KY612958.1, KY612957.1, KY612956.1, KY612955.1, KY612954.1, KY612953.1, KY612952.1, KY612951.1, KY612950.1, KY612949.1, KY612948.1, KY612947.1, KY612946.1, KY612945.1, KY612944.1, KY612943.1, KY612942.1, KY612941.1, KY612940.1, KY612939.1, KY612938.1, KY612937.1, KY670721.1, MF449136.1, KX827589.1, KX827588.1, KX216756.1, KX216755.1, KX216754.1, KX216753.1, KX216752.1, KX216751.1, KX216750.1, KX216749.1, KX216748.1, KX216747.1, KX216746.1, KX216745.1, KX216744.1, KX216743.1, KX216742.1, KX216741.1, KX216740.1, KX216739.1, KX216738.1, KX216737.1, KX216736.1, KX216735.1, KX216734.1, KX216733.1, EU863775.1, EU863774.1, EU863773.1, EU863772.1, EU863771.1, EU863770.1, EU863769.1, EU863768.1, EU863767.1, EU863766.1, EU863765.1, EU863764.1, EU863763.1, EU863762.1, EU863761.1, EU863760.1, EU863759.1, EU863758.1, EU863757.1, EU863756.1, EU863755.1, EU863754.1, EU863753.1, EU863752.1, EU544168.1, EU544167.1, EU544166.1, EU544165.1, EU544164.1, EU544163.1, EU544162.1, EU544161.1, EU544160.1, EU544159.1, EU544158.1, EU544157.1, EU544156.1, EU544155.1, EU544154.1, EU544153.1, EU544152.1, EU544151.1, DQ456998.1, DQ456997.1, DQ003279.1, AY539855.1, AF464901.1, JN118617.1, HQ231797.1, GU646416.1, GU646415.1, GU646414.1, GU646413.1, GU646412.1, GU646411.1, GU646410.1, GU646409.1, GU646408.1, GU646407.1, GU646406.1, GU646405.1, GU646404.1, HM131224.1, HM131222.1, GU299811.1, GU299810.1, AY321957.1, AY321956.1, AY321955.1, AY321954.1, AY321953.1, AY321952.1, AY321951.1, AY321950.1, AY321949.1, GU172372.1, GU172371.1, GU172370.1, GU135651.1, GQ985444.1, GQ985443.1, GQ985442.1, GQ985441.1, GQ985440.1, GQ985439.1, GQ985438.1, GQ985437.1, GQ985436.1, GQ985435.1, GQ866120.1, GQ866119.1, FJ497058.1, FJ497057.1, KU532142.1, KU532141.1, KU532140.1, KU532139.1, KU532138.1, KU532137.1, KU532136.1, KU532135.1, KU532134.1, KU532133.1, DQ916276.1, DQ916275.1, DQ916274.1, DQ916273.1, DQ916272.1, DQ916271.1, DQ916270.1, DQ916269.1, DQ916268.1, DQ916267.1, DQ916266.1, DQ916265.1, DQ916264.1, DQ916263.1, DQ916262.1, DQ916261.1, DQ916260.1, DQ916259.1, DQ916258.1, DQ916257.1, DQ916256.1, DQ916255.1, DQ916254.1, DQ916253.1, DQ916252.1, DQ916251.1, DQ916250.1, DQ916249.1, DQ916248.1, DQ916247.1, DQ916246.1, DQ916245.1, DQ916244.1, DQ916243.1, DQ916242.1, DQ916241.1, DQ916240.1, DQ916239.1, DQ916238.1, DQ916237.1, DQ916236.1, DQ916235.1, DQ916234.1, DQ916233.1, DQ916232.1, DQ916231.1, DQ916230.1, DQ916229.1, DQ916228.1, DQ916227.1, DQ916226.1, DQ916225.1, DQ916224.1, DQ916223.1, DQ916222.1, DQ916221.1, DQ916220.1, DQ916219.1, DQ916218.1, DQ916217.1, DQ916216.1, DQ916215.1, DQ916214.1, DQ916213.1, DQ916212.1, DQ916211.1, DQ916210.1, DQ916209.1, DQ916208.1, DQ916207.1, DQ916206.1, DQ916205.1, DQ916204.1, DQ916203.1, DQ916202.1, DQ916201.1, DQ916200.1, DQ916199.1, DQ916198.1, DQ916197.1, DQ916196.1, DQ916195.1, DQ916194.1, DQ916193.1, DQ916192.1, DQ916191.1, DQ916190.1, DQ916189.1, DQ916188.1, DQ916187.1, DQ916186.1, DQ916185.1, DQ916184.1, DQ916183.1, DQ916182.1, DQ916181.1, DQ916180.1, DQ916179.1, DQ916178.1, DQ916177.1, DQ916176.1, DQ916175.1, DQ916174.1, DQ916173.1, DQ916172.1, DQ916171.1, DQ916170.1, DQ916169.1, DQ916168.1, DQ916167.1, DQ916166.1, DQ916165.1, DQ916164.1, KT633995.1, KR604888.1, KR604887.1, KR604886.1, KR604885.1, KR604884.1, KR604883.1, KR604882.1, KM262810.1, KM205793.1, KJ699103.1, KJ625007.1, KJ625006.1, KJ625005.1, KJ625004.1, KJ625003.1, KJ625002.1, KJ625001.1, KJ625000.1, KJ624999.1, KF444834.1, KF444833.1, KF444832.1, KF444831.1, KF444830.1, KF444829.1, KF444828.1, KF444827.1, KF444826.1, KF444825.1, KF444824.1, KF006251.1, KF006250.1, KC985247.1, KC918850.1, KC918849.1, KC918848.1, KC918847.1, KC918846.1, KC918845.1, KC918844.1, KC918843.1, KC918842.1, KC918841.1, KC918840.1, KC918839.1, KC918838.1, KC918837.1, KC918836.1, JX983160.1, FJ615508.1, FJ615507.1, FJ615506.1, FJ615505.1, FJ615504.1, FJ615503.1, FJ615502.1, FJ615494.1, JX429996.1, JX429995.1, JX429994.1, JX429993.1, JX429992.1, JX429991.1, JX429990.1, JX429989.1, JX429988.1, JX429987.1, JX429986.1, JX429985.1, JX429984.1, JX429983.1, JX429982.1, JX429981.1, JX429980.1, JX429979.1, JX429978.1, JX429977.1, JX429976.1, HQ452820.1, HQ452819.1, HQ452818.1, HQ452817.1, HQ452816.1, HQ452815.1, HQ452814.1, FJ615519.1, FJ615518.1, FJ615517.1, FJ615516.1, FJ615515.1, FJ615514.1, FJ615513.1, FJ615512.1, FJ615511.1, FJ615510.1, FJ615509.1, FJ615501.1, FJ615500.1, FJ615499.1, FJ615498.1, FJ615497.1, FJ615496.1, FJ615495.1, JQ340024.1, JQ340023.1, JQ340022.1, JQ340021.1, JQ340020.1, JQ340019.1, JQ340018.1, JQ340017.1, JQ340016.1, JQ340015.1, JQ260887.1, JQ260886.1, JQ260885.1, JQ260884.1, JQ260883.1, JQ260882.1, JQ260881.1, JQ260880.1, JQ260879.1, JQ260878.1, JQ260877.1, JQ260876.1, JQ260875.1, JQ260874.1, JQ260873.1, JQ260872.1, JQ260871.1, JQ260870.1, FJ168022.1, FJ168021.1, FJ168020.1, FJ168019.1, FJ168018.1, FJ168017.1, FJ168016.1, DQ003289.1, DQ003288.1, DQ003287.1, DQ003286.1, DQ003285.1, DQ003284.1, DQ003283.1, DQ003282.1, DQ003281.1, DQ003280.1, DQ003278.1, AF281312.1, AF281311.1, JQ411012.1, OM307063.1, MZ888508.1, AB605259.1, AF363389.1, OP056767.1, OP056766.1, OP056765.1, OP056764.1, OP056763.1, MT892934.1, MT892933.1, MF043592.1, DQ381813.1, DQ381812.1, DQ381811.1, DQ381810.1, AF121256.1, AY332562.1, AY332561.1, AY332560.1, AF262030.1, AF248612.1, KF021490.1, AY624999.1, FJ824672.1, AJ621158.1, AJ577092.1, OR206436.1, OR206435.1, OR206434.1, OR206433.1, OR206432.1, OR206431.1, OR206430.1, OR206429.1, OR206428.1, OR206427.1, OR206426.1, OR206425.1, AY704912.1, OQ828704.1, OQ828703.1, OQ828702.1, OQ828701.1, OQ828700.1, OQ828699.1, OK032601.1, MN702848.1, MN702847.1, MN702846.1, MN702845.1, MN702844.1, MN702843.1, MN702842.1, MN702841.1, MN702840.1, MN702839.1, MN702838.1, MN702837.1, MN702836.1, MN702835.1, MN702834.1, MN702833.1, MN702832.1, MN702831.1, MN702830.1, MN702829.1, MN702828.1, MN702827.1, MN702826.1, KY938034.1, KY938033.1, KY938032.1, KY938031.1, KY938030.1, AY850693.1, AY739669.1, EF070181.1, EF070180.1, EF070179.1, EF070178.1, EF070177.1, EF070176.1, EF070175.1, EF070174.1, EF070173.1, EF070172.1, EF070171.1, EF070170.1, EF070169.1, EF070168.1, EF070167.1, EF070166.1, EF070165.1, EF070164.1, EF070163.1, EF070162.1, EF070161.1, EF070160.1, EF070159.1, EF070158.1, EF070157.1, AY780423.1, AY780422.1, AY780421.1, AY780420.1, AY780419.1, AY780418.1, AY769979.1, AY769978.1, D10065.1 , OR791879.1, OR791878.1, OR791877.1, OR791876.1, OR791875.1, OR791874.1, OR791873.1, OR506940.1, OR506939.1, OR506938.1, OR506937.1, OR506937.1, OR506935.1, OR506934.1, OR506933.1, OR506932.1, OR506931.1, OR506930.1, OR506929.1, OR506928.1, OR506927.1, OR506926.1, OR506925.1, OR506924.1, OR506923.1, OR506922.1, OR506921.1, OR506920.1, OQ616944.1, OQ616943.1, MZ687402.1, MZ687401.1, MZ687400.1, MZ687399.1, MZ687398.1, MZ687397.1, MZ687396.1, MZ687395.1, MZ766405.1, MZ766404.1, MZ766403.1, MZ766402.1, MZ766401.1, MZ766400.1, MZ766399.1, MZ766398.1, MZ766397.1, MZ766396.1, MZ766395.1, MZ766394.1, MZ766393.1, MZ766392.1, MZ766391.1, MZ766390.1, MZ766389.1, MZ766388.1, MZ766387.1, MZ766386.1, MZ766385.1, MZ766384.1, MZ766383.1, MZ766382.1, MZ766381.1, MW925052.1, MW925051.1, MT550879.1, MT550878.1, MT550877.1, MT550876.1, MT550875.1, MT087553.1, MT087552.1, MT087551.1, MT087550.1, MT087549.1, MT087548.1, MT087547.1, MT215087.1, MT215086.1, MT215085.1, MT215084.1, MT215083.1, MT215082.1, MT215081.1, MT215080.1, MT215079.1, MT215078.1, MT215077.1, MT215076.1, MT215075.1, MT215074.1, MT215073.1, MT215072.1, MN782240.1, EU082025.1, EU082024.1, MK116894.1, MK116893.1, MK116892.1, MK116891.1, MK116890.1, MK116889.1, MK116888.1, MK116887.1, MG944835.1, MG944834.1, KY555598.1, KY555596.1, KY555594.1, KY555592.1, KY555590.1, KY555588.1, KY555588.1, KY555584.1, KY555582.1, KY555580.1, KY555578.1, KY555576.1, KY555574.1, KY555572.1, KY555570.1, AY963143.1, AY963142.1, AY963141.1, AY963140.1, AY963139.1, AY963138.1, AY963137.1, AY963136.1, AY963135.1, AY963134.1, AY963133.1, AY963132.1, AY963131.1, AY963130.1, AY963129.1, AY963128.1, AY963127.1, AY963126.1, AY963125.1, AY963124.1, AY963123.1, AY963122.1, AY963121.1, AY963120.1, AY963119.1, AY963118.1, AY963117.1, AY963116.1, AY963115.1, AY963114.1, AY963113.1, AY963112.1, AY963111.1, AY963110.1, AY907014.1, AY907013.1, AY907012.1, AY907011.1, AY907010.1, AY907009.1, AY907008.1, AY907007.1, AY907006.1, AY907005.1, AY907004.1, AY907003.1, AY907002.1, AY907001.1, AY907000.1, AY906999.1, AY906998.1, AY906997.1, AY739668.1, AY739667.1, AY739666.1, AY520911.1, AY520910.1, AY520909.1, AY423560.1, EF397240.1, EF397239.1, EF397238.1, EF397237.1, KP742967.1, KF573194.1, KF573193.1, KF573192.1, KF573191.1, M64285.1 , JF812065.1, JF812064.1, AB368950.1, AB368949.1, AB368948.1, AB368947.1, AB368946.1, AB368945.1, AB368944.1, AB368943.1, AB368942.1, AB368941.1, OM046618.1, OM046617.1, OM046616.1, OM046615.1, OM046614.1, OM046613.1, OM046612.1, OM046611.1, OM046610.1, OM046609.1, OM046608.1, OM046607.1, OM046606.1, OM046605.1, OM046604.1, OM046603.1, OM046602.1, OR711912.1, OR471614.1, OR471613.1, OR471612.1, OR465344.1, OR465343.1, OR465342.1, OR465341.1, OR465340.1, OR465339.1, OR465338.1, OR465337.1, OR465336.1, OR465335.1, OR465334.1, OR465333.1, OR465332.1, OR465331.1, OR465330.1, OR465329.1, OR465328.1, OR465327.1, OR465326.1, OR465325.1, OR465324.1, OR465323.1, OR465322.1, OR465321.1, OR465320.1, OR465319.1, OR465318.1, OR465317.1, OR465316.1, OR465315.1, OR465314.1, OR465313.1, OR465312.1, OR465311.1, OR465310.1, OR465309.1, OR465308.1, OR465307.1, OR465306.1, OR465305.1, OR465304.1, OR465303.1, OR465302.1, OR361708.1, OR361707.1, OR361706.1, OR361705.1, OR002155.1, OQ938859.1, OQ915152.1, ON661790.1, ON661789.1, ON661788.1, ON661787.1, ON661786.1, ON661785.1, ON661784.1, ON661783.1, ON661782.1, ON661781.1, ON661780.1, ON661779.1, ON661778.1, ON661777.1, ON661776.1, ON661775.1, ON661774.1, ON661773.1, ON661772.1, ON661771.1, ON661770.1, ON661769.1, ON661768.1, ON661767.1, ON661766.1, ON661765.1, ON661764.1, ON661763.1, ON661762.1, ON661761.1, ON661760.1, ON661759.1, ON661758.1, ON661757.1, ON661756.1, ON661755.1, ON661754.1, ON661753.1, ON661752.1, ON661751.1, ON661750.1, ON661749.1, ON661748.1, ON661747.1, ON661746.1, ON661745.1, ON661744.1, ON661743.1, ON661742.1, ON661741.1, ON661740.1, ON661739.1, ON661738.1, ON661737.1, ON661736.1, ON661735.1, ON661734.1, ON661733.1, ON661732.1, ON661731.1, ON661730.1, ON661729.1, ON661728.1, ON661727.1, ON661726.1, ON661725.1, ON661724.1, ON661723.1, ON661722.1, ON661721.1, ON661720.1, ON661719.1, ON661718.1, ON661717.1, ON661716.1, ON661715.1, ON661714.1, ON661713.1, ON661712.1, ON661711.1, ON661710.1, ON661709.1, ON661708.1, ON661707.1, ON661706.1, ON661705.1, ON661704.1, ON661703.1, ON661702.1, ON661701.1, ON661700.1, ON661699.1, ON661698.1, ON661697.1, ON661696.1, ON661695.1, ON661694.1, ON661693.1, ON661692.1, ON661691.1, ON661690.1, ON661689.1, ON661688.1, ON661687.1, ON661686.1, ON661685.1, ON661684.1, ON661683.1, ON661682.1, ON661681.1, ON661680.1, ON661679.1, ON661678.1, ON661677.1, ON661676.1, OL901632.1, OL901631.1, OL901630.1, OL901629.1, OL901628.1, OL901627.1, OL901626.1, OL901625.1, OL901624.1, OL901623.1, OL901622.1, OL901621.1, OL901620.1, OL901619.1, OL901618.1, OL901617.1, OL901616.1, OL901615.1, OL901614.1, OL901613.1, OL901612.1, OL901611.1, OL901610.1, OL901609.1, OL901608.1, OL901607.1, OL901606.1, ON464183.1, OM310975.1, OM310974.1, OM310973.1, OM310972.1, OM310971.1, MZ667872.1, MZ667871.1, MT840280.1, MT840279.1, MT840276.1, MT840275.1, MT840274.1, MT840273.1, MT840272.1, MT840271.1, MT840269.1, MT840266.1, MT840265.1, MT840263.1, MW452665.1, MT304670.1, MT304669.1, MT304668.1, MN700903.1, MN782241.1, AY065637.1, AY065635.1, AY065632.1, AY065631.1, AY065630.1, MN049990.1, MN480452.1, KY200662.1, DQ355820.1, DQ355818.1, DQ355817.1, DQ355815.1, DQ202329.1, GQ856677.1, GQ856676.1, GQ856676.1, GQ856674.1, GQ856673.1, GQ856672.1, GQ856671.1, GQ856670.1, GQ856669.1, GQ856668.1, GQ856667.1, GQ856666.1, EF544169.1, EF419773.1, EF419772.1, EF419771.1, AF293804.1, AF293803.1, AF293802.1, AF293801.1, AF293800.1, AF293799.1, AF293798.1, AF293797.1, AF293796.1, AF293795.1, AF293794.1, AF293793.1, AF293792.1, AF293791.1, AF293790.1, AF293789.1, AF293788.1, AF293787.1, AF293786.1, AF293785.1, AF293784.1, AF293783.1, AF293782.1, AF293781.1, AF293780.1, AF293779.1, AF293778.1, AF293777.1, AF293776.1, AF293775.1, AF293774.1, KU530211.1, KU530210.1, KU530209.1, KU530208.1, KR338954.1, KP836446.1, KP777820.1, KP777819.1, |

**Supplementary Table 4:** Summary of the tools, functions, parameters, and thresholds used in the study.

| **S/N** | **Tools** | **Function** | **Link** | **Parameters for Threshold** | **Threshold** |
| --- | --- | --- | --- | --- | --- |
| 1 | National Centre for Biotechnology Information (NCBI) | To retrieve protein sequences | https://www.ncbi.nlm.nih.gov/protein | - | - |
| 2 | Vaxijen v2.0 Server | To determine the antigenicity of the referenced sequence | http://www.ddg-pharmfac.net/VaxiJen/VaxiJen/VaxiJen.html | - | 0.52 |
| 3 | NetMHCpan-4.1 server | To predict Cytotoxic T-cell Lymphocyte (CTL) epitopes | https://services.healthtech.dtu.dk/service.php?NetMHCpan-4.1 | Threshold for strong binder (% Rank):1  Threshold for weak binder (% Rank): 5 | IC50 |
| 4 | MixMHC2 pred server | To select the highest quality MHC class I CLA alleles | http://ec2-18-188-210-66.us-east-2.compute.amazonaws.com:4000/# | - | - |
| 5 | AllerTop v2.0 | To assess the allergenicity of the epitopes | https://www.ddg-pharmfac.net/AllerTOP/ | - | Defaul-t |
| 6 | ToxinPred 2 server | To predict the toxicity of the epitopes | https://webs.iiitd.edu.in/raghava/toxinpred2/ | Hybrid (RF+BLAST+MERCI) | Default (0.6) |
| 7 | NetMHCIIPan-4.0 | To predict Helper T- cell Lymphocytes (HTL) epitopes that can bind to MHC class II HLA alleles | https://services.healthtech.dtu.dk/services/NetMHCIIpan-4.0/1-Submission.php | - | 0.40 |
| 8 | IFN-γ epitope tool | To predict the ability of the epitopes to induce interferon gamma (IFN-γ) cytokine | http://crdd.osdd.net/raghava/ifnepitope/ | Motif and SVM hybrid  IFN-gamma versus Non IFN-gamma | Default |
| 9 | IL4pred | To predict the ability of the epitopes to induce interleukin 4 (IL-4) | http://crdd.osdd.net/raghava/il4pred/predict.php | - | - |
| 10 | IL10 pred | To predict the ability of the epitopes to induce interleukin 10 (IL-10) | http://crdd.osdd.net/raghava/il10pred/predict.php | - | - |
| 11 | ABCpred | To analyze the antigenic consensus sequences so as to find antigens that can stimulate the B-cell immune response | <http://crdd.osdd.net/raghava/abcpred/> | Threshold [ 0.1 to 1 ]  Window length to use for prediction:  16 | 0.70 |
| 12 | BEPIpred | To analyze the antigenic consensus sequences in order to find antigens that can stimulate the B-cell immune response | <http://tools.iedb.org/bcell/> | - | 0.50 |
| 13 | ElliPro server | To observe the solvent exposure and flexibilities, as well as the linear and structural forms of the epitopes | http://tools.iedb.org/ellipro/ | - | - |
| 14 | IEDB population coverage tool | To estimate the population size that is immune responsive to the constructed vaccine | http://tools.iedb.org/population/ | - | - |
| 15 | Expasy Protparam server | To determine the physicochemical properties of the construct | https://web.expasy.org/protparam/ | - |  |
| 16 | ProteinSol server | To determine the solubility of the construct. | https://protein-sol.manchester.ac.uk/ | - | - |
| 17 | SOPMA | To significantly improve the secondary structure prediction of the mRNA vaccine | <https://npsa-prabi.ibcp.fr/cgi-bin/npsa_automat.pl?page=/NPSA/npsa_sopma.html> | Number of conformational states: 4(Helix, Sheet, Turn  Similarity threshold :8  Window width: 17 | Default |
| 18 | AlphaFold2 | For the prediction of the tertiary structure of the vaccine construct | [https://colab.research.google.com/github/sokrypton/ColabFold/blob/main/AlphaFold2.ipynb#scrollTo=G4yBrceuFbf3](https://colab.research.google.com/github/sokrypton/ColabFold/blob/main/AlphaFold2.ipynb) | - | - |
| 19 | GalaxyRefne server | To enhance the quality of the structure, the prediction of tertiary structure of the designed protein vaccine | <https://galaxy.seoklab.org/cgi-bin/submit.cgi?type=REFINE> | - | - |
| 20 | SAVES 2.0 server | To validate the model vaccine | <https://saves.mbi.ucla.edu/> | - | - |
| 21 | ProSA-web server | To analyze the stereochemical properties of the model vaccine | <https://prosa.services.came.sbg.ac.at/prosa.php> | - | - |
| 22 | HDock server | To study the interactions between the refined model of the tertiary construct with toll-like receptors (TLRs) | http://hdock.phys.hust.edu.cn/ | - | - |
| 23 | UniprotKB server | To retrieve the three-dimensional structures of the selected toll-like receptors (TLRs) | https://www.uniprot.org/ | - | - |
| 24 | Water simulation tool | To study the physical basis of the structure and function of the complex | https://simlab.uams.edu/index.php | - | - |
| 25 | C-ImmSimm server | To ascertain the interaction between the vaccine construct and the host immune system | https://kraken.iac.rm.cnr.it/C-IMMSIM/ | - | - |
